# Transcriptome Sequence Analysis Elaborates a Complex Defensive Mechanism of Grapevine (*Vitis vinifera* L.) in Response to Salt Stress

**DOI:** 10.3390/ijms19124019

**Published:** 2018-12-12

**Authors:** Le Guan, Muhammad Salman Haider, Nadeem Khan, Maazullah Nasim, Songtao Jiu, Muhammad Fiaz, Xudong Zhu, Kekun Zhang, Jinggui Fang

**Affiliations:** 1 College of Horticulture, Nanjing Agricultural University, Nanjing 210095, China; guanle@njau.edu.cn (L.G.); salman.hort1@gmail.com (M.S.H.); 2016104235@njau.edu.cn (N.K.); maazullah.nasim@gmail.com (M.N.); fiaz.m2002@gmail.com (M.F.); zhuxudong@njau.edu.cn (X.Z.); 2006204006@njau.edu.cn (K.Z.); 2 Department of Plant Science, School of Agriculture and Biology, Shanghai Jiao Tong University, Shanghai 200240, China; 2013104019@njau.edu.cn

**Keywords:** grapevine, salt stress, ROS detoxification, phytohormone, transcription factors

## Abstract

Salinity is ubiquitous abiotic stress factor limiting viticulture productivity worldwide. However, the grapevine is vulnerable to salt stress, which severely affects growth and development of the vine. Hence, it is crucial to delve into the salt resistance mechanism and screen out salt-resistance prediction marker genes; we implicated RNA-sequence (RNA-seq) technology to compare the grapevine transcriptome profile to salt stress. Results showed 2472 differentially-expressed genes (DEGs) in total in salt-responsive grapevine leaves, including 1067 up-regulated and 1405 down-regulated DEGs. Gene Ontology (GO) and Kyoto Encyclopedia of Genes and Genomes (KEGG) annotations suggested that many DEGs were involved in various defense-related biological pathways, including ROS scavenging, ion transportation, heat shock proteins (HSPs), pathogenesis-related proteins (PRs) and hormone signaling. Furthermore, many DEGs were encoded transcription factors (TFs) and essential regulatory proteins involved in signal transduction by regulating the salt resistance-related genes in grapevine. The antioxidant enzyme analysis showed that salt stress significantly affected the superoxide dismutase (SOD), peroxidase (POD), catalase (CAT) and glutathione S-transferase (GST) activities in grapevine leaves. Moreover, the uptake and distribution of sodium (Na^+^), potassium (K^+^) and chlorine (Cl^−^) in source and sink tissues of grapevine was significantly affected by salt stress. Finally, the qRT-PCR analysis of DE validated the data and findings were significantly consistent with RNA-seq data, which further assisted in the selection of salt stress-responsive candidate genes in grapevine. This study contributes in new perspicacity into the underlying molecular mechanism of grapevine salt stress-tolerance at the transcriptome level and explore new approaches to applying the gene information in genetic engineering and breeding purposes.

## 1. Introduction

Grapevine (*Vitis vinifera*) is an economic fruit crop, primarily categorized into the table (fresh) and wine grapes [1]. Recent shifts in the environment have become the critical limiting factors for yield and grapevine products. Thus, it is indispensable to characterize the salt-tolerant grapevine varieties by screening salt resistance-related genes and genetically transform them to enable plants to withstand high salt concentrations. One-fifth of irrigated agricultural lands are affected by soil salinity, which leads to escalating the salt effects on plant growth investigations in the recent few years [2,3,4]. High soil salinity affects plants in multiple ways, such as inhibition of water uptake in the root zone, which makes it difficult for the plants to take up water; and results in dehydration of plant cells, leading to cell turgor and in response, plants have to increase osmotic pressure in their cells [5]. Also, due to the decrease in K^+^/Na^+^ value, the original balance of ions in plant cells might be interrupted, which has a toxic effect on enzymes, chlorophyll degradation and recurrent protein synthesis [6]. Simultaneously, salt induces cellular toxicity, which leads to undue reactive oxygen species (ROS) production and accumulation in different cellular compartments, resulting in lipid peroxidation (LPO) of biological membranes, ions leakage and DNA-strand cleavage [7].

Plants can evolve a complex defensive mechanism to counteract the salinity effects [8], which includes activation of numerous signaling sensors that conclusively excites various transcription factors (TFs) to induce stress-responsive genes, which enable plants to nurture and transcend the adverse conditions. In salinity, factors involved in signaling are: (i) discerning accretion or elimination of ions to stabilize the K^+^/Na^+^ balance and other ion levels via salt-inducible enzyme Na^+^/H^+^ antiporter (V-ATPase or PPase) and K^+^ and Na^+^ transporters (SOS family); (ii) biosynthesis of congenial solutes to adjust the vacuolar ionic balance and restore water in the biochemical reaction (Like polyols and mannitol); (iii) adjust the cell membrane structure; (iv) synthesis of multiple resistance-oriented proteins like ROS and pathogenesis-related proteins (PR family); and (v) induction of plant hormones (ABA, JA and IAA). These biological pathways improve the inclination of salt tolerance are likely to collaborate and may have the synergistic effect [6,9]. Besides, various transcription factors (TFs), such as HD-Zip, ERF, WRKY, bHLH are known to play a vital role in regulating salt resistance mechanism in plants [1,10].

Recently, next-generation sequencing (NGS) technology based high throughput RNA-seq technology has been extensively used to unveil and compare the transcriptome profile under abiotic stresses [1], which provides large-scale data to identify and characterize the DEGs. Previously, extensive studies have been carried out on antioxidant metabolism, ionomic uptake and transport, hormonal metabolism and stress signaling [11,12] but the underlying molecular mechanism of salt stress tolerance remain to be elucidated. Though several studies focusing on morphological variations, biochemical and physiological components are available in grapevine, however, there is no report on transcriptomic studies particularly molecular research associated with salt stress tolerance. Therefore, to comprehend the molecular mechanism of salt tolerance in grapevine, Illumina RNA-seq libraries were constructed from both control and salt-treated grapevine leaves. In addition, gene ontology (GO) enrichment analysis was also performed to investigate biochemical and physiological cues in response to salt stress. qRT-PCR analysis of critical salt stress-responsive genes was also carried out to validate RNA-seq results. The obtained information provides more profound insights into the grapevine molecular mechanism in improving breeding strategies for the development of transgenic plants, which can better resist the abiotic stress.

## 2. Results

### 2.1. Global Transcriptome Sequence Analysis

The transcriptomic sequencing of cDNA generated from both control and salt-treated grapevine leaf samples produced 21.2 and 21.4 million raw reads, respectively (Table S1). Following the filtering and trimming process, 20.2 and 20.6 million clean reads were retrieved from control and treatment group, respectively, corresponding to 8.16 Gb data, intimating the tag density from both control and salt-treatment, representing about 20 million reads, which is adequate for quantitative analysis of gene expression. For sequence alignments, SOAPaligner/soap2 software (http://soap.genomics.org.cn) was used as reference genome of grapevine (Version 1.0), suggesting total mapped reads as 67.4% matched complemented with both unique (57.42%) or multiple (9.96%) genomic positions (Table S1).

Transcriptome analysis can compare the number of DEGs and their expression pattern in different tissues. In our transcriptomic study, 21,746 transcripts were obtained from control and 21,541 transcripts from the treatment group. Among these expressed transcripts, 14,767 transcripts showed no significant changes in their expression level (|log_2_ FC)| < 1), while 2472 transcripts were differentially expressed in the salt-treatment group (|log_2_ FC)| ≥ 1) at false discovery rate (FDR) <0.001), which includes 1067 (43.16%) up-regulated and 1405 (56.87%) down-regulated transcripts (Table S2). Moreover, 20 DEGs suggested their expression only in the control group and 27 DEGs were only expressed in the treatment group (Table S3).

### 2.2. GO and KEGG Analysis of DEGs in Response to Salt Stress

**Figure 1:**
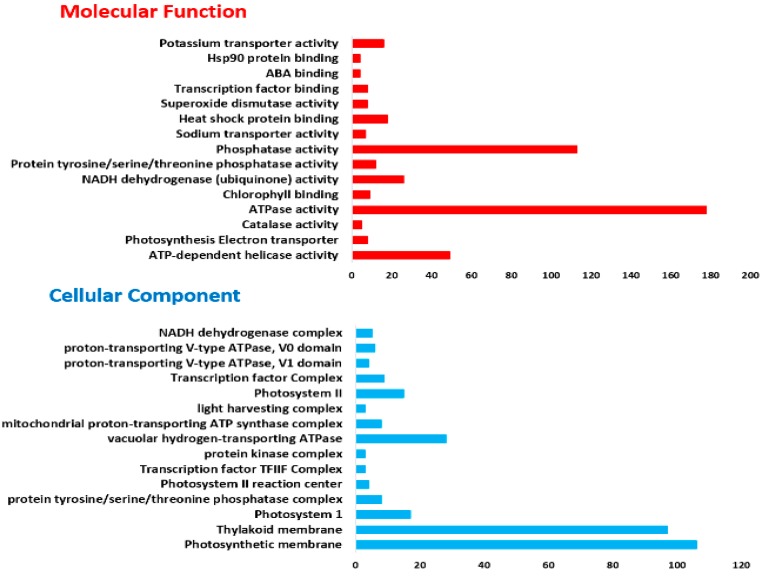

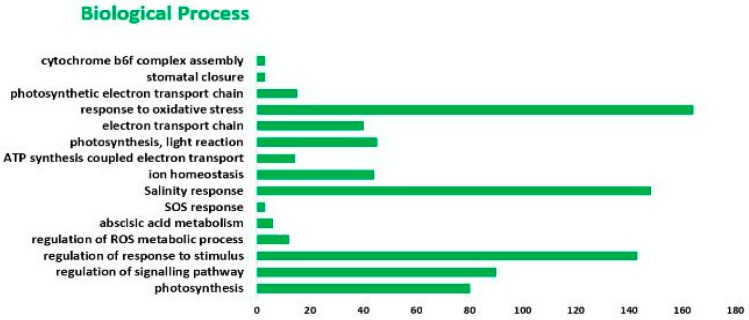
Gene ontology (Go) based annotations of 2472 DEGs. The main GO terms are categorized into “molecular function”, “cellular component” and “biological process”.

GO-based enrichment analysis functionally characterizes and annotated the 1, 591 (64.36% of 2, 472) DEGs into 45 functional groups, of which molecular function contains 15 groups, cellular component (15 groups) and biological process (9) (Figure 1 and Table S4) between control and salt-treated group. In molecular function (MF), “ATPase activity” (GO: 0042623) with 178 transcripts, followed by “phosphatase activity” (GO: 0008138) with 113 transcripts and least transcripts (4) were found in both “ABA binding (GO: 0010427)” and “Hsp90 protein binding (GO: 0010329)”. In cellular component (CC), “photosynthetic membrane” possessed the highest number of transcripts (GO: 0034357, 106 transcripts), whereas, “thylakoid membrane” consisted of 97 transcripts (GO: 0042651). Furthermore, in biological process (BP), “response to oxidative stress” (GO: 0006979) harbored 164 transcripts, followed by “salinity response” (GO: 0009651) with 148, while “SOS response (GO: 0009432)”, “stomatal closure (GO: 0090332)” and “cytochrome b6f complex (GO: 0010190)” with three transcripts each were the least group.

**Figure 2:**
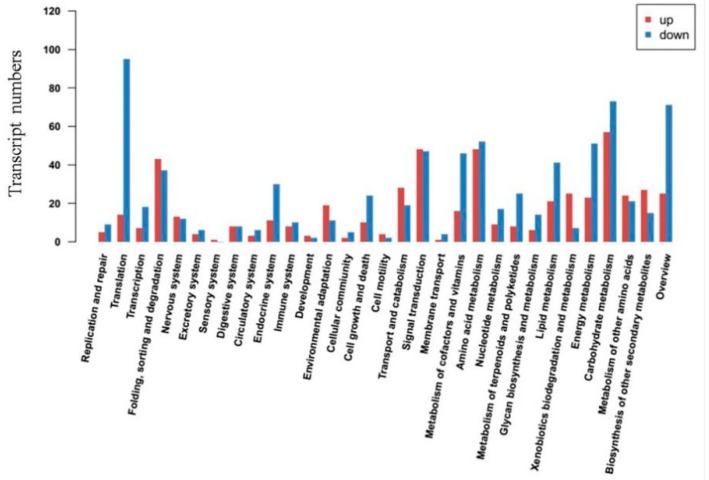
Kyoto Encyclopedia of Genetics and Genomics (KEGG) database analysis of DEGS (up and down-regulated) enriched in different biological pathways. The *X*-axis represents enriched pathways and *Y*-axis represents the total number of transcripts.

KEGG database simulates the functional annotation of the cells or the organism by sequence similarity and genome information. In this study, 453 (18.32% of 2472) transcripts were allocated to 30 pathways in KEGG database (Figure 2 and Table S5), while “Signal transduction” pathway with 79 transcripts was the most enriched pathway followed by “Folding, sorting and degradation” (65 transcripts) and “Carbohydrate metabolism” (63 transcripts).

### 2.3. Photosynthetic Efficiency of Grapevine in Response to Salt Stress

**Figure 3:**
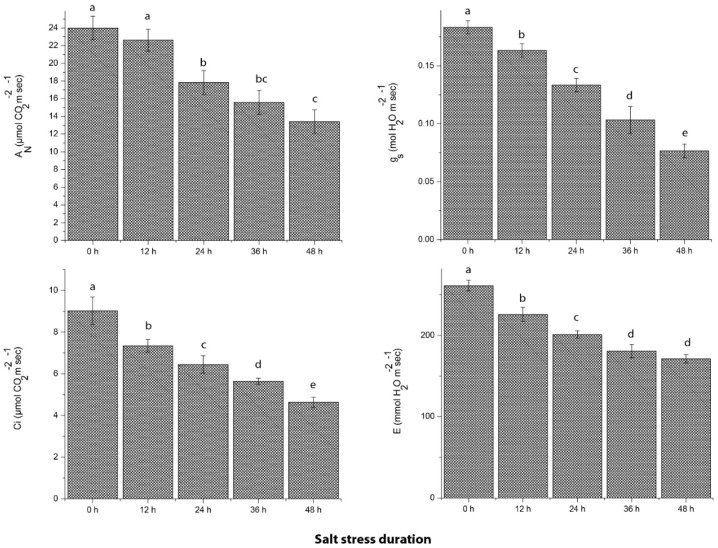
Estimation of photosynthetic efficiency, including net photosynthesis rate (A_N_), stomatal conductance (g_S_), transpiration rate (E) and net CO_2_ assimilation rate (Ci) in grapevine leaves in response to salt stress as compared with control. Values represent mean ± SE (*n* = 3) and the significance level of 0.05 was used for different letters above bars.

To verify the extent of salt severity on grapevine physiology, photosynthetic efficiency and related parameters were estimated in the control and treatment group by using a portable Li-COR meter. Results suggested that net photosynthesis rate (A_N_) was significantly reduced from 23.98 ± 1.33 (0 h) to 13.42 ± 1.31 (48 h) during the salt stress period. Likewise, an about 2-fold decrease in stomatal conductance significantly inhibited the net CO_2_ assimilation rate (Ci; 35.78%) and transpiration rate (E; 51.33%) after 48 h of salt stress as compared to control plants (Figure 3). In the transcriptomic study, the DEGs encoding photosystem II CP47 (psbB) in PSII and photosystem I P700 (psaB) in PSI were down-regulated in salt-treated grapevine leaf samples as compared to control (Table S2), which is consistent with the physiological investigations of decreased net photosynthesis rate. Moreover, six DEGs encoding ATP-synthase and one DEG-related to the cytochrome b6-f complex were also down-regulated in grapevine leaf tissues after exposure to salt stress.

### 2.4. Production and Scavenging of Reactive Oxygen Species (ROS) in Response to Salt Stress

ROS production is a universal plant response to almost all type of abiotic stresses. In response, plants accumulate various antioxidant enzymes (SOD, POD and CAT) that can quench free radicals, such as H_2_O_2_ and O_2_^•−^ [12]. In this study, 44 DEGs were identified as enzymes in the ROS detoxification and scavenging system. These DEGs were functionally characterized into different ROS enzymes encoding Fe superoxide dismutase (Fe-SODs, 1 transcript), catalase (CAT, 2 transcripts), peroxidase (POD, 8 transcripts), glutathione S-transferase (GST, 16 transcripts), alternative oxidase (AOX, 1 transcript), glutathione-ascorbate (GSH-AsA) cycle (6 transcripts), the peroxiredoxin/thioredoxin (Prx/Trx, 9 transcripts) and polyphenol oxidase (PPO, 1 transcript) (Table 1 and Table S6).

**Figure 4:**
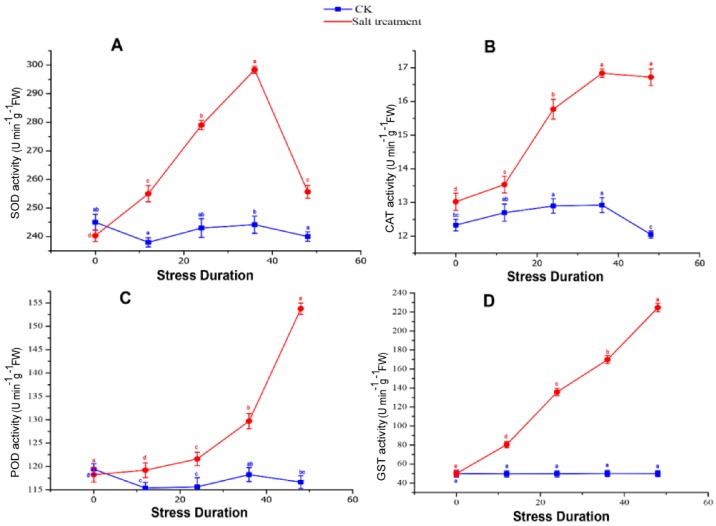
Changes in the enzyme activities of SOD (**A**), CAT (**B**), POD (**C**) and GST (**D**) in grapevine leaves grown for 48 h under control and salt stress. Values represent mean ± SE (*n* = 3) and the significance level of 0.05 was used for different letters above bars.

The metalloenzyme superoxide dismutase (SOD) provides primary defense line against ROS (superoxide radicals, O_2_^•−^) and dismutates O_2_^•−^ into O_2_ and H_2_O_2_. SODs have three isozymes that are localized in different cellular compartments and vary in their functional properties, including copper-zinc (Cu/Zn-SOD), manganese (Mn-SOD) and iron (Fe-SOD). While only one Fe-SOD with slightly down-regulated expression level (|log_2_ FC| > 1) was found in this research, might be due to the severity of salt that suppressed the transcription of Fe-SOD gene in grapevine leaves. These findings are consistent with the previous reports [13,14,15] and were also confirmed by the SOD activity measurement, in which SOD activity was increased within 36h of salt stress but drastically decreased after 48 h (Figure 4a). In current findings, the activities of CAT and POD were progressively persuaded at 48 h of salt stress treatment (Figure 4b,c). Transcriptomics analysis showed that the DEGs encoding CAT and POD were up-regulated under salt stress, of which two POD transcripts, VIT_13s0067g02360 (|log_2_ FC| = 3.68) and VIT_08s0040g02200 (|log_2_ FC| = 3.51) were remarkably up-regulated in salt-treated group as compared to control, while remaining six POD and the two CAT transcripts were slightly up-regulated (their |log_2_ FC| values were about 1), which is consistent with the physiological data of increased activities of antioxidant enzymes. These findings suggested a common response of antioxidant enzymes to detoxify ROS effects.

GSH and GST also play a crucial defense-related role against ROS caused by salt stress [16,17]. In this study, six transcripts involved in ascorbate-glutathione (AsA-GSH) cycle and 16 Glutathione S-transferase (GST) transcripts were detected, of which salt stress significantly induced two GST transcripts (VIT_05s0049g01070 and VIT_05s0049g01100), when compared with remaining four genes. GST activity was also significantly increased at 48 h of salt stress (Figure 3d), revealing its essential roles in the ROS scavenging process.

### 2.5. Heat Shock Protein (HSP) and Pathogenesis-Related Proteins (PR) in Response to Salt Stress

Heat shock proteins (HSPs) are the molecular chaperones that act as stress-responsive proteins, thus protecting plants from stress damage, which include mainly HSP100s, HSP90s, HSP70s, HSP60s (cpn60s) and small heat-shock proteins (sHSPs). Overall, 39 HSPs-related DEGs were further divided into high molecular weight HSPs (HMW HSPs; 4 transcripts), low molecular weight HSPs (LMW HSPs; 17 transcripts), heat stress transcription factors (6 transcripts) and other HSPs (12 transcripts) (Table 2 and Table S7). Three transcripts encoding HMW HSPs were down-regulated, while one transcript (VIT_18s0041g01230) was up-regulated. Similarly, 16 of the 17 LMW HSPs were up-regulated and some of them showed very high expression levels compared to the control, for example, VIT_16s0098g01060 (|log_2_ FC| = 3.755743188), VIT_13s0019g00860 (|log_2_ FC| = 3.361853346) and VIT_12s0035g01910 (|log_2_ FC| = 3.135505213), intimating that LMW HSPs play a more important role than the HMW HSPs in response to salt stress in grapevine. Six heat stress TFs (3 up-regulated and 3 down-regulated) showed very diverse transcription levels, suggesting their complex regulatory mechanism over HSPs. Also, 8 chaperone protein DnaJ (6 up-regulated and 2 down-regulated) transcripts identified in the current research, as DnaJ is a vital cofactor plays a central role in transducing stress-induced protein damage to induce heat shock gene transcription, while the up-regulation may suggest the extensive cellular protein damage by salt severity.

Plants can enhance tolerance mechanism against salt stress through over-expression of pathogenesis-related proteins. In the grapevine transcriptome, 37 DEGs encoding disease resistance proteins were identified and classified into 4 pathogenesis-related proteins (PR-1; all up-regulated), 2 chitinase (both down-regulated), 1 beta-1, 3-glucanase (down-regulated), 8 lipid transfer proteins (7 up-regulated, 1 down-regulated), 6 thaumatin-like proteins (1 up-regulated, 5 down-regulated), 1 germin protein (down-regulated), 13 disease resistance proteins (9 up-regulated, 4 down-regulated) and 2 snakin were perceived as up-regulated (Table 2 and Table S7).

### 2.6. Regulation of Hormonal Signaling in Response to Salt Stress

Hormones are pivotal to plants in stress adaptive signaling cascades and act as a central integrator to connect and reprogram different responses, such as photosynthesis and activities of ROS enzymes, protein structure and gene expression and accumulation of secondary metabolites [18,19,20]. In this experiment, various DEGs encoding hormone signaling was involved in abscisic acid (ABA), jasmonic acid (JA), auxin (IAA), gibberellin (GA), ethylene (ETH), brassinosteroid (BR) synthesis and signal transduction pathways (Table S8). Under salt stress, ABA is known to play a protective role in plants against LPO by assisting the accumulation of metabolites that act as osmolytes and also tends to close their stomata to reduce water loss by transpiration. Moreover, 8 transcripts encoding protein phosphatase 2C (PP2C) were down-regulated in the salt-treated grapevine leaves, while PP2C is deliberated as a negative regulator of the ABA signaling. Also, 2 ABA receptor PYL (1 up and 1 down-regulated) were also detected, which indicated that salt stress-induced not only the regulators but also the receptors in the ABA transduction pathway, by which ABA signaling pathway was enhanced quickly and then participated in the salt stress defense process.

Other plant hormones, like auxin and ethylene, also have important roles in plants to cope with salt stress. In this experiment, 23 auxin-related transcripts were detected, in which 2 auxin response factors (ARF) and 3 auxin-responsive proteins were down-regulated, while 12 auxin-induced proteins and 3 indole-3-acetic acid-induced proteins were up-regulated. Out of the 12 auxin-induced proteins, 4 transcripts (VIT_04s0023g00530, VIT_03s0038g01100, VIT_03s0038g01090 and VIT_04s0023g00520) were only expressed in the salt-treated samples, suggesting their close interaction with salt stress. In ethylene synthesis, 3 ACC oxidases (ACO) homologs were up-regulated, whereas 23 transcripts encoding ethylene-responsive TFs revealed variation in up-regulation (13 transcripts) and down-regulation (10 transcripts) in grapevine under salt stress.

### 2.7. Ion Transport Systems Mediating Na^+^ Homeostasis in Response to Salt Stress

**Figure 5:**
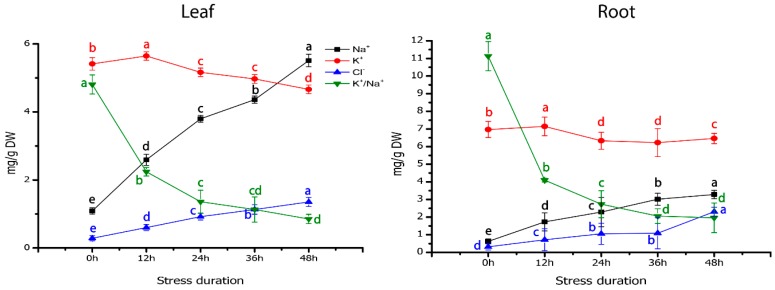
Ion concentrations of sodium (Na^+^), potassium (K^+^), chlorine (Cl^−^) and K^+^/Na^+^ ratio in leaf and root samples of grapevine grown for 48 h of salt stress. Values represent means ± SE (*n* = 3) and the significance level of 0.05 was used for different letters above bars.

Salt tolerance mechanism works basically by reducing the undue accretion of Na^+^ in the cytosol of the plant cell. The quantification of ionic concentrations suggested that Na^+^ concentration increased significantly in leaf and root tissues (Figure 5). Leaves had higher Na^+^ accumulation (5.51 ± 0.48), which was 40.47% more than Na^+^ level of roots (3.28 ± 0.23). Moreover, Cl^−^ concentration increased significantly at about 5-folds in leaves and 9-folds in roots as compared to their corresponding controls, respectively. In contrast, K^+^ showed a decreasing trend in both tissues (leaf and root) after 48h of salt stress as compared with the control group (Figure 5). In the transcriptomic analysis, 14 DEGs were found to be involved in the ion transport systems, which include 2 vacuolar-type H^+^-ATPase (V-type proton ATPase, 1 up and 1 down-regulated), 2 sodium/hydrogen exchanger (both down-regulated), 3 cyclic nucleotide-gated ion channel (CNGC, 2 up-regulated and one down-regulated), 2 potassium transporter (both down-regulated), 2 K^+^ efflux antiporter (both down-regulated), 2 sodium-related cotransporter (sodium/pyruvate cotransporter, sodium/bile acid cotransporter (down-regulated) transcripts (Table S9). In the vacuolar membrane, V-ATPase is the central H^+^ pump, which creates a transmembrane proton gradient and drives the Na^+^/H^+^ antiporter to transport the excessive Na^+^ in the cytoplasm to vacuoles [21].

### 2.8. Transcription Factors in Response to Salt Stress

**Figure 6:**
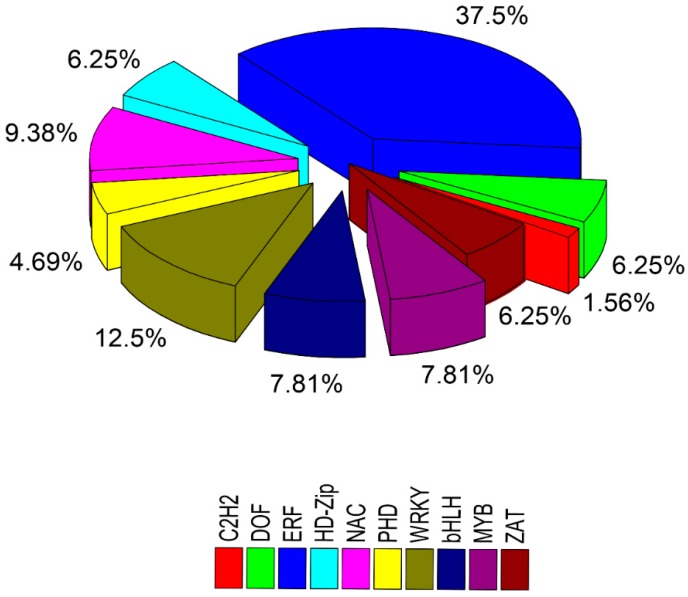
The sector diagram of major TFs identified and the total number of DEGs in grapevine leaf tissues after 48 h of salt stress compared with control.

Transcription factors (TFs) are proteins that cooperate with other transcriptional regulators and bind cis-elements at the promoter region, thus up-regulate the downstream activities of many stress-related genes, results in inducing stress resistance in plants. Almost all the TFs identified in the present transcriptome data have already been reported to play a significant role to counter salt stress (Table S10). Results revealed five MYB transcripts (4 up-regulated, 1 down-regulated), 8 WRKY transcripts (all down-regulated), 1 C2H2 transcript (down-regulated), 4 DOF transcripts (3 up-regulated, 1 down-regulated), 6 HD-zip transcripts (all up-regulated), 5 bHLH transcripts (1 up-regulated and 4 down-regulated), 4 ZAT transcripts (1 up-regulated, 3 down-regulated), 6 NAC transcripts (1 up-regulated and 5 down-regulated), 3 PHD transcripts (all up-regulated) and 23 ERF TFs, intimating their critical roles in the grapevine resistance to salt stress (Figure 6).

### 2.9. qRT-PCR Validation of Illumina RNA-Seq Results

**Figure 7:**
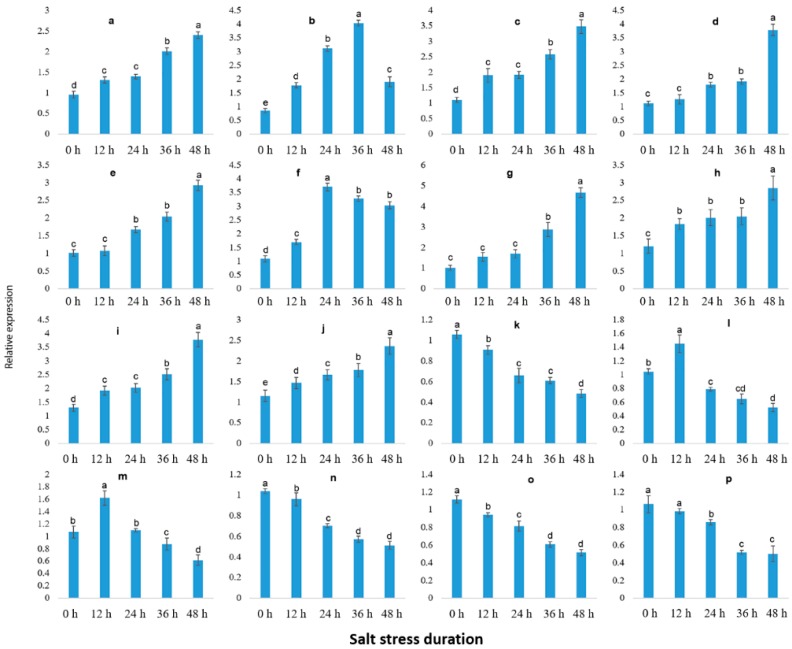
qRT-PCR validation of illumina Hiseq findings and screening of damage severity prediction marker genes. Values represent means ± SE (*n* = 3) and the significance level of 0.05 was used for different letters above bars. Genes have continual increasing or decreasing expression patterns were selected as candidate genes. **a**: VIT_05s0020g03740.t01, **b**: VIT_16s0050g02530.t01 **c**: VIT_19s0015g01070.t01, **d**: VIT_05s0049g00520.t01, **e**: VIT_13s0067g02360.t01, **f**: VIT_12s0035g01910.t01, **g**: VIT_04s0023g00530.t01, **h**: VIT_05s0049g01070.t01, **i**: VIT_06s0004g05670.t01, **j**: VIT_10s0003g01810.t01, **k**: VIT_00s0332g00110.t01, **l**: VIT_00s0201g00080.t01, **m**: VIT_07s0005g00160.t01, **n**: VIT_11s0052g01180.t01, **o**: VIT_14s0128g00020.t01, **p**: VIT_05s0062g00300.t01.

To validate the reliability of RNA-seq transcriptome, 16 DEGs were randomly selected to analyze the gene expression that was correlated with salt stress response and covering almost all the primary functions in various biological pathways, including transcription factors, metabolism, plant hormone signaling, disease resistance and ion transport (Table S11). The result suggested that expression of 16 DEGs treated with 0.8% soil salinity at the interval of 0, 12, 24, 36 and 48 h is inconsistent with the transcriptomic findings, validating the accuracy and reproducibility of the Illumina RNA-seq. Though, out of 16 DEGs, 12 DEGs showed recurrent expression pattern in response to salt stress, in which 8 genes were up-regulated (Figure 7a,c–e,g–j) and 4 genes were down-regulated (Figure 7k,n–p) with prolonged salt stress. Based on the expression patterns of these 12 genes, we selected them as candidate genes to further validate their expressional variations following the different concentrations of salt stress and recovery process.

### 2.10. Salt Stress Recovery and the Selection and Validation of Marker Genes 

**Figure 8:**
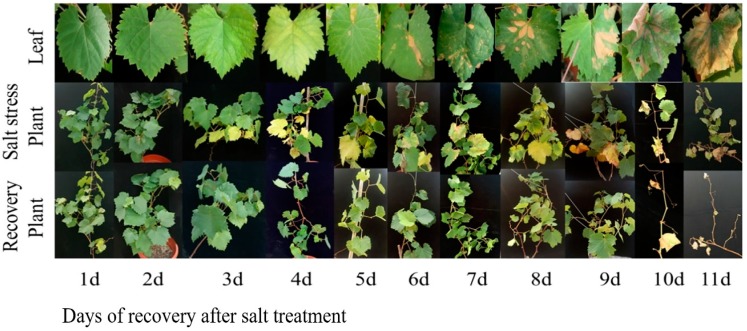
Grapevine growth status under salt stress and after removing salt stress. Grapevine plants were treated by 1.5% SS (salt stress) for 1, 2, 3,4, 5, 6, 7, 8, 9, 10 and 11 days (d), respectively and recovered by washing away the salt in the medium. Recovered plants were photographed 15 days after salt stress was removed.

**Figure 9:**
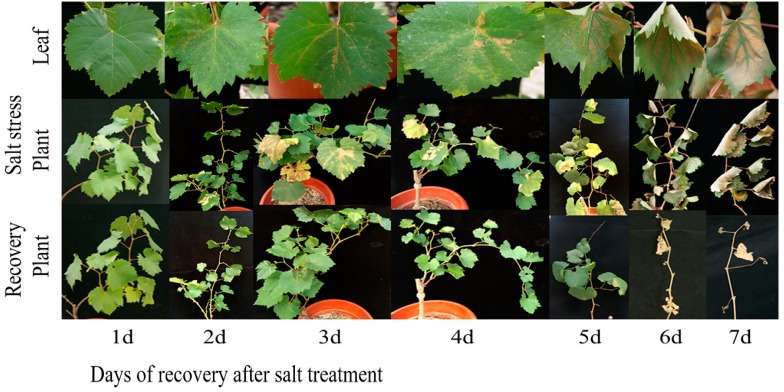
Grapevine growth status under salt stress and after removing salt stress. Grapevine plants were treated by 3.0% SS for 1, 2, 3, 4, 5, 6 and 7 days (d), respectively and recovered by washing away the salt in the medium. Recovered plants were photographed 15 days after salt stress was removed.

**Figure 10:**
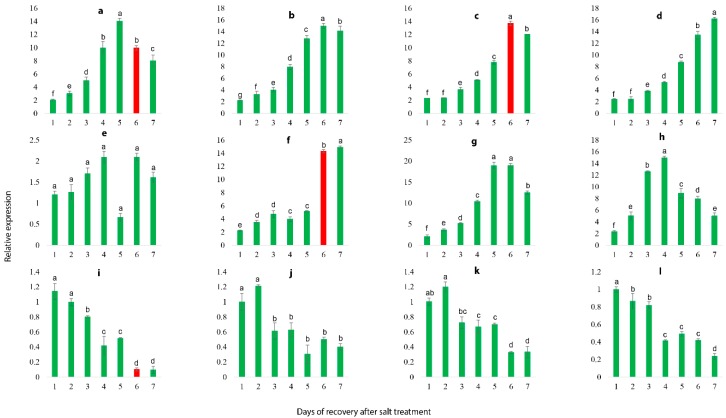
Expression patterns of the 12 candidate genes following the 7 days of 1.5% SS. Red bars indicate the sharp change in gene expression levels. Values represent mean ± SE (*n* = 3) and the significance level of 0.05 was used for different letters above bars. Genes with continual increasing or decreasing expression patterns were selected as candidate genes. **a**: VIT_05s0020g03740.t01, **b**: VIT_19s0015g01070.t01, **c**: VIT_05s0049g00520.t01, **d**: VIT_13s0067g02360.t01, **e**: VIT_04s0023g00530.t01, **f**: VIT_05s0049g01070.t01, **g**: VIT_06s0004g05670.t01, **h**: VIT_10s0003g01810.t01, **i**: VIT_07s0005g00160.t01, **j**: VIT_11s0052g01180.t01, **k**: VIT_14s0128g00020.t01, **l**: VIT_05s0062g00300.t01.

Herein, 12 marker genes showing regular expression patterns, defined their potential as useful markers to determine the stress severity in grapevine plants. The growth status of grapevine plants was monitored, which indicated that salt severity turned grapevine leaves yellow and brownish blemishes were developed after a prolonged duration of salt stress and eventually die (Figure 8 and Figure 9). At 1.5% salt concentration, grapevine plants can be recovered to normal growth conditions within 10 days of salt treatment by removing the salt stress, though few injured leaves could not survive even after the recovery, might be due to over-accumulation of salt. On the contrary, the plants died after prolonged salt stress duration (15 days), though there was no phenotypic evidence of death before going for recovery. Likewise, the critical time of recovery for 3.0% salt stress is 6 days. Similarly, qRT-PCR analysis of 12 candidate genes showed increased/ decreased expression level following the different doses of salt application. Furthermore, some genes showed unique expression pattern following 10 days of stress, such as VIT_05s0020g03740 showed an increasing trend within 9 days of salt stress but decreased significantly on the 10th day of salt stress (Figure 10a); whereas, the expression level of VIT_05s0049g00520, VIT_05s0049g01070 and VIT_06s0004g05670 was induced within 9 days after stress but sharply induced on the 10th day of salt stress (Figure 10d,h,i). Nevertheless, two transcripts (VIT_00s0332g00110 and VIT_05s0062g00300) showed a gradual decrease in their expression level till the 9th day but the sharp decrease was observed at the 10th day after NaCl application (Figure 10k,p).

**Figure 11:**
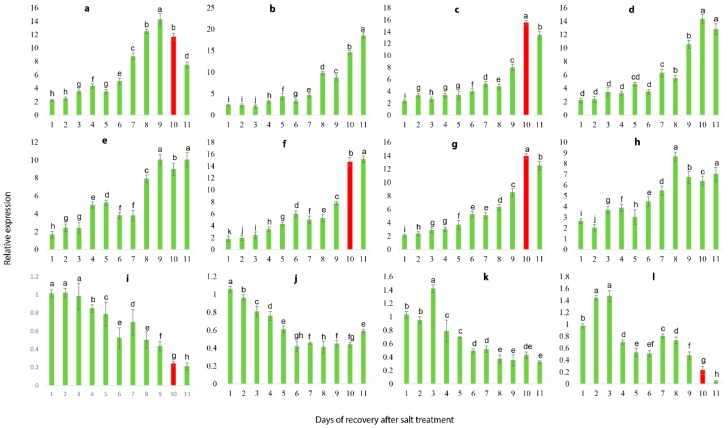
Expression patterns of the 12 candidate genes following the 11 days of 3.0% SS. Red bars indicate the sharp change in gene expression levels. Values represent mean ± SE (*n* = 3) and the significance level of 0.05 was used for different letters above bars. Genes with continual increasing or decreasing expression patterns were selected as candidate genes. **a**: VIT_05s0020g03740.t01, **b**: VIT_19s0015g01070.t01, **c**: VIT_05s0049g00520.t01, **d**: VIT_13s0067g02360.t01, **e**: VIT_04s0023g00530.t01, **f**: VIT_05s0049g01070.t01, **g**: VIT_06s0004g05670.t01, **h**: VIT_10s0003g01810.t01, **i**: VIT_07s0005g00160.t01, **j**: VIT_11s0052g01180.t01, **k**: VIT_14s0128g00020.t01, **l**: VIT_05s0062g00300.t01.

Interestingly, some of these genes showed a similar sharp expression level at the 6th day under 3.0% salt stress, such as transcript VIT_05s0020g03740 kept increasing until 5th day of stress but suddenly decreased on the 6th day of salt stress (Figure 11a), while the expression levels of VIT_05s0049g00520 and VIT_05s0049g01070 kept slow increasing trends up to 5 days of salt stress but showed a sharp increase on the 6th day (Figure 11d,h). Moreover, transcript VIT_00s0332g00110 showed a gradually decreasing trend up to 5 days of salt stress but significantly reduced on the 6th day of salt stress (Figure 11k). Based on above-mentioned findings, grapevine plants cannot be survived by curative processes after 10 days at 0.8% of NaCl and after 6 days at 1.5% of NaCl, which indicates that regardless of high or low concentrations of salt, these four genes with recurrent expression pattern could be used as potential markers to predict the severity imposed by salt stress.

## 3. Discussion

Salt stress is considered as most severe abiotic stress, which impairs all principal physiological functions, including photosynthesis, lipid metabolism and synthesis of proteins [22]. To confront the stress, plants are compelled to initiate protective responses, like restoring cellular ion concentrations and reducing the toxicity of ions like Na^+^/H^+^, K^+^ and Cl^−^. Moreover, the accretion of osmoprotectants and hydrophilic proteins, such as sugars, polyols, proline, glycine betaine (GB), amino acids (AA) and amines are crucial for governing the osmotic potential pressure. Also, the accumulation of ROS enzymes and antioxidants is vital to prevent tissue damage by eliminating the free radicals induced by salt stress [22,23].

Grapevine plants alter their physiology to combat salt stress severity. Current findings suggested that reduced stomatal conductance resulted in the inhibition of net photosynthesis rate and CO_2_ exchange, which is considered as a primary response of grapevine to reduce transpiration rate to avoid salt accumulation in stomatal apertures of leaves. Zhang et al. [24] depicted that stress factors damage the photosynthetic pigments (chlorophylls and carotenoids) in both photosystems (PSI and PSII), which affect their light-absorbing efficiency, resulting in hindered photosynthetic capability. Our results are consistent with the findings of similar work on peach [25] and grapes [1], proposing that photosynthetic efficiency and CO_2_ balance were affected by the reduced stomatal conductance. Moreover, the light-harvesting proteins (CP47) in PSII and chlorophyll binding proteins (P700) in PSI were down-regulated by the salt stress. Similar study intimated that salt stress induces ROS production, which damages the LHCs in PSI and impairs the PSII proteins involved in the evolution of oxygen [26]. Also, the modifications in leaf biochemistry decrease the synthesis of ATP amount, leading to regeneration of the RuBISCO, which results in down-regulation of photosynthetic metabolism [27], favor our findings of down-regulation of RuBISCO and ATP-related transcripts. In *Arabidopsis*, oxidative stress activates SnRK2 in ABA signaling, which regulates the stomatal conductance [27] and up-regulation of SnRK21 in our findings might be the reason for the inhibited photosynthetic activity of grapevine leaves.

Salt stress affects the large-scale metabolic activities that result in excessive ROS accumulation, which include singlet oxygen (^1^O_2_), superoxide radical (O_2_^•−^), hydrogen peroxide (H_2_O_2_) and hydroxyl radical (•OH), while similar results were observed in *Medicago truncatula* [28]. The ROS cytotoxicity activates the oxygen species, leading to disruption of optimum metabolic activities, which induce lipid peroxidation in plants [29,30]. Thus, the equilibrium between ROS production and quenching is critical under salt stress. Plants can unfold a complex antioxidative defense system to limit the oxidative damage, which mainly comprised of enzymatic antioxidant (SOD, CAT, POD and GST) and non-enzymatic antioxidants (AsA, GSH, proline and phenolic compounds) [31,32]. In the present study, the antioxidative defense system was activated in salt-treated leaves, although SOD-related transcripts were down-regulated, while CAT, POD and GST-related transcripts were significantly up-regulated. Similar research on *Pyrus pyrifolia* [33] and *Fagopyrum tataricum* [34] depicted that over-expression of GST transcripts significantly enhances salt stress tolerance. Moreover, the enhanced activities of ROS enzymes (CAT, POD and GST) are consistent with the transcriptomic data, which symbolize their vital functions in ROS detoxification. However, CAT activity increased significantly in our findings till 36 h of salt stress but drastically decrease at 48 h, which is in agreement with the down-regulation of SOD-related transcripts in grapevine under salt stress. Zhang et al. [35] reported that salt stress up-regulates the expression of CAT, POD and GST and increases the corresponding enzymes activities. Similarly, the complex accumulation pattern of antioxidant enzyme activities was observed in our findings, which is consistent with the findings of grapevine [36] and soybean [37] under salt stress.

HSPs are the molecular chaperones known to participate in the translocation and degradation of damaged proteins under abiotic stresses [38,39]. In the current study, HSP70 and HSP90 were down-regulated, while various heat stress TFs, small HSPs (sHSPs16–30 kDa) and other HSPs, like DnaJ, were up-regulated by salt stress. This irregular trend of HSPs may suggest that HSPs play an adaptive stress role by altering the growth and development of the plant. Several homologs of HSPs were also found to be activated in *Betula halophila* and *F. tataricum* under salt stress, intimating their regulatory role in various signaling-related pathways [34,40]. The disease resistance proteins can protect plants from pathogens by infection-induced responses of the immune system [41]. In our study, most pathogenesis-related proteins, non-specific lipid-transfer protein and disease resistance proteins were remarkably up-regulated, signifying that these genes not only function in disease resistance but also play essential roles in plant responses to salt stress [42].

Ionic compartmentalization and absorption are essential for growth under saline conditions because stress disrupts ion homeostasis [43]. Plant roots uptake Na^+^ and other ions with water from the soil and translocate these ions to the leaves via transpiration stream. With the evaporation of water, a high level of salt gets accumulated in the apoplast and other cellular compartments. Ionic imbalance induces cellular toxicity via replacement of K^+^ by Na^+^ ions via interfering K^+^ channels in the plasma membrane of the root [9,44], while all the potassium transporters were down-regulated in our findings and resulted in lower K^+^ concentration in the salt-treated group as compared to control. The plant can resist the cytosolic salt accumulation in the vacuole and other cellular compartments to facilitate their metabolic functions [45,46]. This process involves the regulation of the expressions of some ionic channels and transporters-related genes, which enables the control of Na^+^ transport within the plant [4,47]. In *Arabidopsis*, vacuolar *AtNa^+^/H^+^* exchanger *SOS1* (Salt Overlay Sensitive) assists Na^+^ extrusion from root cells [48,49] but Na^+^/H^+^ exchanger was down-regulated in our findings, which might be the reason of Na^+^ accumulation in the grapevine roots. In addition, NHX1 was down-regulated in our results, while *AtNHX1* cloned plants resulted in high Na^+^ in shoot tissues by altering the gene expression of Na^+^ transporters [50]. The transcriptional activation of vacuolar-type ATPase (V-ATPase) in our findings suggested that it assists plants in reducing Na^+^ accumulation by interacting H^+^ pumps to counter salt stress [51]. The high-affinity K^+^ transporters (HKTs) can mediate Na^+^ transport and Na^+^–K^+^ symport, while the over-expressed *Arabidopsis AtHKT1* showed high Na^+^ in leaves and reduced accumulation in roots [52], favor our findings of higher Na^+^ accumulation in grapevine leaf tissues as compared to root. The inhibition of Na^+^ influx is the correlative index of cyclic nucleotide-gated ion channels CNGCs that were up-regulated in this study, which is in good agreement with the findings reported in halophyte shrub (*Nitraria sibirica*) [53]. The genetic factors that control the accumulation and transport of Cl^−^ from root to leaf tissues or enable plants to maintain low leaf Cl^−^level are the critical determinant of salt stress tolerance in plants [54], while higher accumulation of Cl^−^ was observed in roots as compared to leaves in our findings, intimating that grapevine possesses the salt tolerance mechanism. However, in response to higher Cl^−^ level, plants harness the Cl^−^/H^+^ transporters (CLCs) to maintain low Cl^−^ accumulation, especially in aerial parts [55], whereas, no gene related to Cl^−^ transport was found in our findings.

Phytohormones create a web of signals that are pivotal to plant growth, initiation of flowers, hypocotyl germination and abiotic stress response, which mainly include abscisic acid (ABA), auxin (AUX), jasmonic acid (JA), brassinosteroid (BR) and ethylene (ETH) [56,57]. High salt concentration triggers the ABA level in many plants, which is a well-known fact [25]. In our study, abscisic acid receptor PYL9 was up-regulated and its negative regulator PP2Cs were down-regulated, proposing that the ABA signaling pathway was activated in grapevine in response to salt stress. However, transcriptomic profiling of Jute (*Corchorus* spp.) revealed that DEGs encoding PYL were down-regulated under salt stress [58], which is contradicting with our findings as well as with the basic model of ABA signaling. Additionally, auxin stimulates cell elongation and cell division, also induces sugar and mineral accumulation at the site of application. Under salt stress, all ARFs and their repressors were down-regulated, whereas, most AUX/IAA proteins and IAA synthase were found up-regulated in our findings, suggesting that genes encoding IAA participate significantly in plant development in response to salt stress conditions, while similar results were reported in *V. vinifera* under oxidative stress [59]. JA generally reconciles specific signaling mechanisms involved in senescence, flowering and defense responses, while all the critical enzymes encoding JA were down-regulated, depicting that gene related to JA were suppressed by the salt severity in *Vitis vinifera*. Another study demonstrated that JA level was enhanced in salt-tolerant cultivars as compared to sensitive cultivars [60]. Salt stress inhibits the cell multiplication and expansion by suppressing the activities of growth-promoting hormones, including gibberellins and cytokinins [60], while these results are in favor of current findings.

TFs are regulatory proteins, demonstrated to be involved in regulating the stress-responsive gene expression in many plants responding to abiotic stress. Various MYB genes have been identified and known to induce plant responses to salt stress acclimation, such as *Arabidopsis* [61], rice [62] and wheat [63]. The over-expression of rice MYBs (*OsMYB48-1* and *OsMYB3R-2*) proposed alleviated tolerance to abiotic stresses, such as salt, cold and drought [64,65]. WRKY gene family is regarded as an essential TFs involved in salt stress response, such as *ZmWRKY33* in maize [66] and *GhWRKY39* in cotton [67], but, in our study, all the eight WRKY transcripts, including two WRKY33 were down-regulated under salt stress, which indicates the complexity of the WRKY regulatory mechanism and diverse nature in different stress conditions. Many other TFs with no direct response to salt stress but were triggered by other physiological changes like ROS and endogenous hormones. ERF family interact with ABA signaling pathway (dependent and/or independent) and respond to abiotic stresses [59,60]. In our study, 23 ERF transcripts were detected in the salt-treated grapevine leaves; meanwhile, ten ABA-related transcripts were also identified, intimating their essential roles in ABA-dependent ERF regulatory mechanism in grapevine. PHD finger proteins, especially PHD2, were reported to be involved in the salt stress response, which is known to induce by salt-induced oxidative stress [68]. All the three PHD transcripts detected in our transcriptome were up-regulated, which suggested the significance ROS synthesis caused by salt stress in the tested samples.

**Figure 12:**
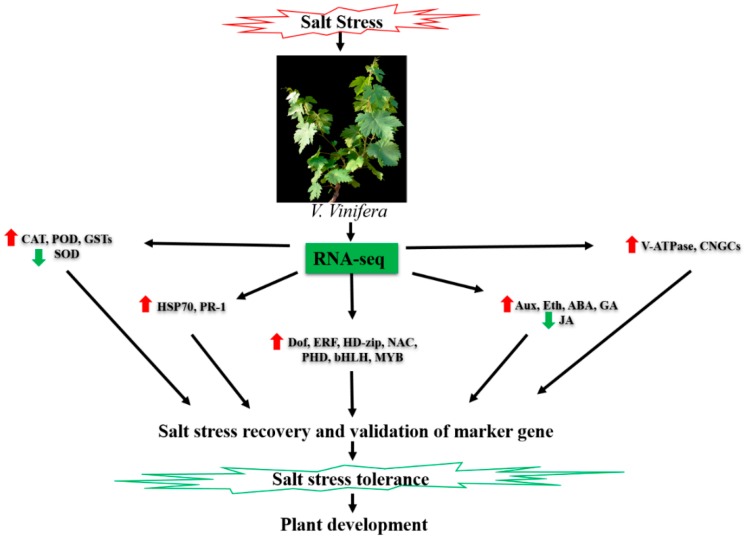
A schematic complex regulatory mechanism of salt stress tolerance in grapevine. Red arrows indicating up-regulated genes and green arrows indicating down-regulated genes. CAT, catalase; POD, peroxidase; GST, glutathione-s-transferase; SOD, superoxide dismutase; HSP70, heat shock protein 70 kDa; PR-1, Pathogenesis-related protein1; Dof, DNA-binding with one finger; ERF, ethylene responsive factor; HD-Zip, homeodomain-leucine zipper; NAC, NAC transcription factor; bHLH, basic helix-loop-helix; MYB, MYB transcription factor; Aux, auxin; Eth, ethylene; ABA, abscisic acid; GA, gibberellic acid; JA, jasmonic acid; V-ATPase, vacuolar-type ATPase; CNGCs, cyclic nucleotide-gated channels.

Specific genes regulate various plant traits and some of these genes expressed in unique patterns before the emergence of apparent traits, thus by detecting these unique expression signals, we can predict the occurrence of the corresponding phenotype. Hence, to measure the stress severity induced by NaCl stress, grapevine plants were subject to salt treatment with different doses of NaCl and time interval and were recovered by washing off the salt from roots. Results suggested that grapevine plants can be recovered within 10 days at 1.5% of NaCl dose and within 6 days under 3% salt stress. Similar expression patterns of genes (VIT_05s0049g00520, VIT_05s0049g01070, VIT_05s0020g03740 and VIT_00s0332g00110) observed in both NaCl treatments (1.5% and 3%) after 10 and 6 days, respectively, which makes them be the marker genes to estimate the salt severity. Selected genes were involved mainly in the maintenance of cellular structure and functions in plants. For instance, gene VIT_05s0020g03740 (non-specific lipid-transfer protein, LTP) and VIT_05s0049g00520 (proline-rich cell wall protein-like, PRPs) are known to play essential roles in maintaining the stability of the cell wall, membrane and osmotic pressure of the cell [69,70,71]. VIT_05s0049g01070 (glutathione S-transferase-like, GST) encoded as a critical protein, which has several physiological functions like ROS detoxification and protecting the DNA from damage [72]. Also, VIT_00s0332g00110 (Photosystem II reaction center protein) was involved in the most important physiological function (photosynthesis). Taken together, the transcriptional status of these marker genes reflects the vitality in grapevine plants. Moreover, a similar technique to predict marker genes can also be implicated on other crops where natural environmental disasters, such as temperature (low and high), water-logging and drought prevails occasionally. Taken together, grapevine possesses a complex regulatory mechanism of salt stress-tolerance, which mainly involves the regulation of key genes that are summarized in Figure 12.

## 4. Materials and Methods

### 4.1. Plant Material and Salt Treatments

Two-year-old grapevine (Summer Black Cv.) pot grown plants obtained from Jiangsu Academy of Agriculture Sciences (JAAS), Nanjing, China and kept in greenhouse conditions (25 ± 5 °C), provided with 65% relative humidity (RH) and 16 h-light and 8 h-dark photoperiod at Nanjing Agricultural University, China. The grapevine plants were kept in a medium of soil-peat-sand at 3:1:1 (*v*:*v*:*v*) and used as experimental materials. Overall, ten grapevine plants were selected and categorized into salt-treated (5 plants) and control (5 plants) groups. NaCl (0.8%) was selected to induce salinity stress in grapevine plants. Fourth-unfolded leaf from both the NaCl-treated and control groups were collected at the interval of 0 (control), 12, 24, 36 and 48 h. Each sample has three replicates. Collected leaf samples were immediately frozen dried in liquid nitrogen and then stored at −80 °C until further analysis.

### 4.2. RNA Extraction, cDNA Library Construction and Illumina Deep Sequencing

Trizol reagent method was used to extract the total RNA from both salt-treated and control grapevine leaf samples (Invitrogen, Carlsbad, CA, USA). The RNA quantity was determined by using Micro-spectrophotometer (Nano-100, ALLSHENG, Hangzhou, China) and further mRNA purification and cDNA library construction were performed with the Ultra™ RNA Library Prep Kit for Illumina (MA, USA) by following the manufacturer’s protocol. The final sampling collected after 48 h from salt treatment was sequenced against control (0 h) on an Illumina HiseqTM2500.

### 4.3. Mapping of reads, Gene Annotation and Analysis of Gene Expression Level 

The raw sequence data were filtered by removing low-quality sequences and adapter reads by using HISAT [1]. After quality trimming, clean reads were mapped to the *V. vinifera* reference genome using Bowtie (1.1.2) by adapting standard mapping parameters [59]. In this data, >100 bp read length with <2 mismatches were mapped to reference genome. To calculate the gene expression and RPKM (and reads per kilobase per million, SAM tools and BamIndexStats.jar were used. Then, DEGseq2 was used to obtain differentially-expressed genes (DEGs) between Log_2_ and the stationary phase [59]. The genes with FDR less than 0.001 and 2-fold change were pondered as DEGs.

### 4.4. Gene Ontology (GO) and Kyoto Encyclopedia of Genes and Genomics (KEGG)

For GO annotations, the DEGs were subjected GO database (http://www.geneontology.org/) by using program Blast2Go (http://www.blast2go.com/ Ver. 2.3.5). To classify genes or their products into terms (molecular function, biological process and cellular component) GO enrichment analysis by using GO-seq was used to under biological functions of DEGs [59]. For KEGG annotations, all the DEGs were mapped to the KEGG database (https://www.genome.jp/kegg/pathway.html) and looked for enriched pathways compared to the background genome [73].

### 4.5. Estimation of Photosynthesis Rate and Determination of Several Enzymatic and Ionic Concentrations

For photosynthesis rate (A_N_), stomatal conductance rate (g_S_), CO_2_ exchange (Ci) and transpiration rate (E), 4th unfolded leaves were used from control and salt-treated grapevine plants between 9:00–11:00 AM, on full sunny day, using portable Li-COR (Li-6400XT, NE, USA) as briefly described by Haider et al. [1].

Leaf samples treated with 0.8% NaCl for 0, 12, 24, 36 and 48 h were used to determine the antioxidative enzymes activities, including SOD, CAT, POD and GST. The activity of SOD was measured using NBT at 560 nm; CAT activity was measured by monitoring disappearance of H_2_O_2_ at 240 nm, the POD was determined by guaiacol oxidation method following the method briefly explained by Haider et al. [74,75]. GST activity was determined using Glutathione S-transferase (GST) activity determination kit (Shanghai solarbio Bioscience & Technology Co., LTD, Shanghai, China) following the manufacturer’s protocol.

For ionic concentrations, 0.5 g of leaf and root sample were first oven dried at 70 °C for 48 h and then ground to powder and digested in HNO3: HClO4 (2:1, *v*:*v*). The concentrations of selected ions (e.g., Na^+^, K^+^ and Cl^−^) were determined using ICP-MS (Thermo Electron Corporation, MA, USA) as previously explained by Ma et al. [76]. The data were subjected to one-way analysis of variance (ANOVA) by using three replicates for each sample and expressed mean ± standard error (SE). Statistical analysis was carried out using Minitab (Ver 16) and SPSS (Ver 15.0) at *p* < 0.05 level of significance.

### 4.6. Quantitative Real-Time PCR (qRT-PCR) Analysis of DEGs and Validation of Illumina RNA-Seq Results

Sixteen genes selected from various pathways were used for the validation of the Illumina RNA-seq by qRT-PCR analysis. The primer pairs were designed using primer3 program (http://bioinfo.ut.ee/primer3-0.4.0/) and details of the primers are shown in supplementary Table S11. After extraction, total RNA was reverse-transcribed using the PrimeScript RT Reagent Kit with gDNA Eraser (Takara, Dalian, China). Each qPCR reaction contains 10 µL 2× SYBR Green Master Mix Reagent (Applied Biosystems, CA, USA), 2.0 µL cDNA sample and 400 nM of gene-specific primer in a final volume of 20 µL.qRT-PCR was carried out using an ABI PRISM 7500 real-time PCR system (Applied Biosystems, CA, USA). PCR conditions were 2 min at 95 °C, followed by 40 cycles of heating at 95 °C for 10 s and annealing at 60 °C for 40 s. A template-free control for each primer pair was set for each cycle. The All PCR reactions were normalized using the *C*t value corresponding to the Grapevine actin gene (XM_010659103). Three biological replications were used and three measurements were performed on each replicate.

### 4.7. Salt Stress and Recovery Assay

To screen out the marker genes following the oxidative stress severity caused by salt, 15 grapevine plants were treated with two different acute salt concentrations (1.5% and 3.0%) and then plants recovered by washing off the NaCl solution. 

Everyday 3 potted grapevine seedlings were recovered from NaCl stress by washing off the salts with the distilled water; this step was repeated till the salinity content from the medium was reduced to the average level (around 0.1%), 1/2 strength of Hoagland nutrient solution with standard NaCl content was watered again. The salt treated plants were sampled and photographed every day during the treatment and recovered plants. All qRT-PCR reactions for the selected marker genes were the same as previously mentioned.

## 5. Conclusions

A comparative transcriptome analysis was explored on two libraries constructed from salt-treated and control grapevine leaf samples. Results revealed that 2472 genes were differentially expressed and were significantly involved in antioxidant system, hormonal signaling, ion homeostasis and disease and pathogenesis-related pathways. Besides, many regulatory proteins encoding transcription factors were also identified that induce the function of other genes (e.g., *HSPs*) requisite for stress-adaptive responses and tolerance. The GO annotations assisted to screen out the series of molecular and physiological cues, which revealed their critical role in salt stress-tolerance mechanism. Moreover, salt stress significantly affected the photosynthetic efficiency and ions uptake and transport in *V. vinifera*. Though, antioxidant enzyme (CAT, POD and GST) activities were enriched to counter the lipid peroxidation. In this study, we have also screened out and validated the four candidate genes to predict salt severity in grapevine. Taken together, current study provided a deep overview of enriched genomic information along with physiological validation that will be useful for understanding the salt stress regulatory mechanism in grapevine.

## Figures and Tables

**Table 1:** List of differentially-expressed genes related to redox metabolism and respiratory chain in grapevine perceived during salt stress.

Trait Name	Description	No. of Up-Regulated	No. of Down-Regulated	Sum
ROS scavenging	Fe-SOD	0	1	1
	POD	8	0	8
	CAT	2	0	2
GSH-AsA cycle	MDAR	1	0	1
	APx	1	0	1
	GR	0	2	2
	Grx	1	1	2
GPX pathway	GST	8	8	16
Prx/Trx	Trx	4	5	9
Cyanide-resistant respiration	AOX	0	1	1
Copper-containing enzymes	PPO	0	1	1

Fe-SOD: Fe superoxide dismutase; POD: peroxidase; CAT: catalase, APX: ascorbate peroxidase; MDAR: monodehydroascorbate reductase; GR: glutathione reductase; Grx: glutaredoxin; GST: glutathione S transferase; Trx: thioredoxin; AOX: alternative oxidase, PPO: polyphenol oxidase.

**Table 2:** List of differentially-expressed genes related to heat-shock proteins (HSPs) and pathogens resistance (PRs) proteins in grapevine perceived during drought stress.

Trait Name	Description	No. of Up-Regulated	No. of Down-Regulated	Sum
Heat shock proteins	HMW HSPs	1	3	4
	LMW HSPs	16	1	17
	small HSPs	12	6	18
	other HSPs	7	5	12
	heat-stress transcription factors	3	3	6
PR-1	pathogenesis-related protein 1	4	0	4
PR-2	β-1,3-glucanase	0	1	1
PR-3,4,8,11	chitinase	0	2	2
PR-5	Thaumatin-like protein	1	5	6
PR-14	lipid transfer protein	7	1	8
PR-15	germin-like protein	0	1	1
	Disease resistance proteins	9	4	13
	snakin	2	0	2

HMW HSPs: High molecular weight heat shock proteins; LMW HSPs: Low molecular weight heat shock proteins.

## Supplementary Materials

Supplementary materials can be found at http://www.mdpi.com/1422-0067/19/12/4019/s1.

## Abbreviations

CAT	Catalase
Cl^−^	Chloride
DEGs	Differentially expressed genes
GO	Gene Ontology
GST	Glutathione S transferase
HSPs	Heat shock proteins
K^+^	Potassium
KEGG	Kyoto Encyclopedia of Genes and Genomes
Na^+^	Sodium
POD	Peroxidase
PRs	Pathogenesis-related proteins
qRT-PCR	Quantitative reverse transcriptome-PCR
ROS	Reactive oxygen species
RNA-seq	RNA-sequencing
SOD	Superoxide dismutase
SS	Salt stress
TFs	Transcription factors
