# Supplementary material for: Transcriptome Sequence Analysis Elaborates a Complex Defensive Mechanism of Grapevine (Vitis vinifera L.) in Response to Salt Stress
Source: Int J Mol Sci. 2018 Dec 12;19(12):4019. doi: 10.3390/ijms19124019 (PMC6321183; doi:10.3390/ijms19124019)
Supplement: Supplementary file 1 [file ijms-19-04019-s001.zip › supplementary tables.docx]

**Additional file 1** Number of reads sequenced and mapped to the grapevine genome.

|  | Control | NaCl treatment | Sum |
| --- | --- | --- | --- |
| Raw Reads | 21236463 | 21426156 | 42662619 |
| Clean Reads | 20230252 | 20568085 | 40798337 |
| Total mapped (percent of clean reads) | 10999416 (54.37%) | 16489341 (80.17%) | 27488757 (67.38%) |
| Unique_match (percent of clean reads) | 9475421 (46.84%) | 13950006 (67.82%) | 23425427 (57.42%) |
| Mutliple_match (percent of clean reads) | 1523995 (7.53%) | 2539335 (12.35%) | 4063330 (9.96%) |
| Total unmapped (percent of clean reads) | 9230836 (45.63%) | 4078744 (19.83%%) | 13309580 (32.62%) |

**Additional file 3** Transcripts expressed only in the NaCl-treated or control group

| #Gene | Length | Function | read_A | | RPKM_A | | read_B | | RPKM_B | | log2 | q-value | p_value | Result |
| --- | --- | --- | --- | --- | --- | --- | --- | --- | --- | --- | --- | --- | --- | --- |
| **expressed only in the salt-treated group** | | | | | | | | | | | | | | |
| T_00s0194g00180.t01 | 637 | rho GTPase-activating protein gacV-like | 156 | | 14.85189 | | 0.5 | | 0.071361 | | 7.701295404 | 1.36E-28 | 9.08E-30 | up |
| VIT_01s0011g05420.t01 | 885 | protein PLANT CADMIUM RESISTANCE 2-like | 141 | | 9.662123 | | 0.5 | | 0.051364 | | 7.555444538 | 3.07E-26 | 2.22E-27 | up |
| VIT_16s0013g00590.t01 | 717 | blue copper protein-like | 117 | | 9.896085 | | 0.5 | | 0.063399 | | 7.286257905 | 2.27E-22 | 1.91E-23 | up |
| VIT_12s0059g02510.t01 | 639 | B-box zinc finger protein 32 | 109 | | 10.34481 | | 0.5 | | 0.071138 | | 7.18407751 | 4.73E-21 | 4.25E-22 | up |
| VIT_15s0046g01690.t01 | 904 | acidic endochitinase-like | 97 | | 6.507288 | | 0.5 | | 0.050284 | | 7.015806028 | 4.89E-19 | 4.85E-20 | up |
| VIT_19s0014g02010.t01 | 585 | UDP-glycosyltransferase 79B9-like | 83 | | 8.604367 | | 0.5 | | 0.077704 | | 6.790932617 | 1.24E-16 | 1.41E-17 | up |
| VIT_04s0023g00530.t01 | 370 | auxin-induced protein 15A | 69 | | 11.30952 | | 0.5 | | 0.122857 | | 6.524417642 | 3.71E-14 | 4.90E-15 | up |
| VIT_09s0002g02950.t01 | 284 | inositol oxygenase 1-like | 67 | | 14.30715 | | 0.5 | | 0.16006 | | 6.481982376 | 8.49E-14 | 1.15E-14 | up |
| VIT_10s0003g01810.t01 | 398 | Putative uncharacterized protein | 65 | | 9.904373 | | 0.5 | | 0.114213 | | 6.438260998 | 1.95E-13 | 2.71E-14 | up |
| VIT_05s0020g03650.t01 | 548 | Putative uncharacterized protein | 64 | | 7.082655 | | 0.5 | | 0.082951 | | 6.415893185 | 2.95E-13 | 4.17E-14 | up |
| VIT_08s0056g00850.t01 | 357 | Putative uncharacterized protein | 61 | | 10.36235 | | 0.5 | | 0.12733 | | 6.346630523 | 1.03E-12 | 1.53E-13 | up |
| VIT_03s0038g01100.t01 | 269 | auxin-induced protein 15A-like | 51 | | 11.4978 | | 0.5 | | 0.168985 | | 6.088318527 | 7.31E-11 | 1.25E-11 | up |
| VIT_03s0038g01090.t01 | 404 | auxin-induced protein X15-like | 49 | | 7.355487 | | 0.5 | | 0.112517 | | 6.03060303 | 1.73E-10 | 3.07E-11 | up |
| VIT_12s0034g01160.t01 | 559 | basic blue protein-like | 47 | | 5.098973 | | 0.5 | | 0.081318 | | 5.970482037 | 4.13E-10 | 7.57E-11 | up |
| VIT_03s0088g00890.t01 | 489 | basic form of pathogenesis-related protein 1 | 41 | | 5.084775 | | 0.5 | | 0.092959 | | 5.77344519 | 5.80E-09 | 1.18E-09 | up |
| VIT_16s0022g01150.t01 | 400 | Putative uncharacterized protein | 35 | | 5.306458 | | 0.5 | | 0.113642 | | 5.545176202 | 8.52E-08 | 1.96E-08 | up |
| VIT_05s0020g03740.t01 | 368 | non-specific lipid-transfer protein-like protein | 33 | | 5.438296 | | 0.5 | | 0.123524 | | 5.460287305 | 2.11E-07 | 5.07E-08 | up |
| VIT_18s0001g01550.t01 | 335 | Putative uncharacterized protein | 32 | | 5.792978 | | 0.5 | | 0.135692 | | 5.415893185 | 3.33E-07 | 8.17E-08 | up |
| VIT_05s0020g03360.t01 | 384 | Putative uncharacterized protein | 32 | | 5.05377 | | 0.5 | | 0.118377 | | 5.415893185 | 3.33E-07 | 8.17E-08 | up |
| VIT_00s0187g00180.t01 | 321 | Putative uncharacterized protein | 28 | | 5.289927 | | 0.5 | | 0.14161 | | 5.223248107 | 2.08E-06 | 5.65E-07 | up |
| VIT_19s0014g02560.t01 | 249 | (-)-germacrene D synthase-like | 26 | | 6.332434 | | 0.5 | | 0.182558 | | 5.116332904 | 5.25E-06 | 1.51E-06 | up |
| VIT_08s0007g01880.t01 | 277 | pentatricopeptide repeat-containing protein | 26 | | 5.692333 | | 0.5 | | 0.164105 | | 5.116332904 | 5.25E-06 | 1.51E-06 | up |
| VIT_04s0023g00520.t01 | 303 | auxin-induced protein 15A | 26 | | 5.203882 | | 0.5 | | 0.150023 | | 5.116332904 | 5.25E-06 | 1.51E-06 | up |
| VIT_17s0000g07770.t01 | 209 | snakin-2-like | 18 | | 5.223035 | | 0.5 | | 0.217497 | | 4.585818187 | 0.00022667 | 8.39E-05 | up |
| VIT_17s0000g04800.t01 | 129 | Putative uncharacterized protein | 18 | | 8.462126 | | 0.5 | | 0.35238 | | 4.585818187 | 0.00022667 | 8.39E-05 | up |
| VIT_13s0064g00350.t01 | 135 | putative disease resistance RPP13-like protein 1 | 16 | | 7.187584 | | 0.5 | | 0.336718 | | 4.415893185 | 0.000590802 | 0.000236 | up |
| VIT_11s0016g03840.t01 | 144 | pentatricopeptide repeat-containing protein （PPR) | 16 | | 6.73836 | | 0.5 | | 0.315673 | | 4.415893185 | 0.000590802 | 0.000236 | up |
| **expressed only in the control group** | | | | | | | | | | | | | | |
| VIT_18s0001g05330.t01 | 66 | Putative uncharacterized protein | 0.5 | 0.459434 | | 10 | | 13.77484 | | -4.906034909 | | 0.000692399 | 0.000281 |  |
| VIT_09s0054g01450.t01 | 99 | Putative uncharacterized protein | 0.5 | 0.306289 | | 10 | | 9.183224 | | -4.906034909 | | 0.000692399 | 0.000281 | down |
| VIT_15s0046g02670.t01 | 126 | Putative uncharacterized protein | 0.5 | 0.240656 | | 11 | | 7.936929 | | -5.043538433 | | 0.000342789 | 0.000131 | down |
| VIT_05s0077g00380.t01 | 138 | Putative uncharacterized protein | 0.5 | 0.219729 | | 12 | | 7.905558 | | -5.169069315 | | 0.000170729 | 6.18E-05 | down |
| VIT_13s0019g00650.t01 | 183 | THO complex subunit 4B | 0.5 | 0.165697 | | 13 | | 6.458366 | | -5.284546533 | | 8.58E-05 | 2.94E-05 | down |
| VIT_00s0615g00040.t01 | 45 | probable mannitol dehydrogenase-like | 0.5 | 0.673836 | | 14 | | 28.28433 | | -5.391461737 | | 4.31E-05 | 1.41E-05 | down |
| VIT_00s0327g00070.t01 | 186 | acyl-[acyl-carrier-protein] desaturase | 0.5 | 0.163025 | | 14 | | 6.842983 | | -5.391461737 | | 4.31E-05 | 1.41E-05 | down |
| VIT_17s0000g03470.t01 | 156 | AMSH-like ubiquitin thiolesterase 1-like | 0.5 | 0.194376 | | 15 | | 8.741723 | | -5.49099741 | | 2.18E-05 | 6.82E-06 | down |
| VIT_19s0085g00470.t01 | 213 | Putative uncharacterized protein | 0.5 | 0.14236 | | 16 | | 6.829214 | | -5.584106815 | | 1.11E-05 | 3.32E-06 | down |
| VIT_05s0077g00640.t01 | 279 | Putative uncharacterized protein | 0.5 | 0.108683 | | 16 | | 5.213701 | | -5.584106815 | | 1.11E-05 | 3.32E-06 | down |
| VIT_07s0005g04320.t01 | 201 | Putative uncharacterized protein | 0.5 | 0.150859 | | 17 | | 7.689237 | | -5.671569656 | | 5.64E-06 | 1.63E-06 | down |
| VIT_02s0012g03100.t01 | 93 | Putative uncharacterized protein | 0.5 | 0.32605 | | 17 | | 16.61867 | | -5.671569656 | | 5.64E-06 | 1.63E-06 | down |
| VIT_00s0256g00100.t01 | 258 | Putative uncharacterized protein | 0.5 | 0.11753 | | 21 | | 7.39997 | | -5.976424237 | | 4.01E-07 | 9.99E-08 | down |
| VIT_14s0030g00820.t01 | 140 | Putative uncharacterized protein | 0.5 | 0.21659 | | 22 | | 14.28647 | | -6.043538433 | | 2.10E-07 | 5.05E-08 | down |
| VIT_13s0175g00010.t01 | 225 | Putative uncharacterized protein | 0.5 | 0.134767 | | 24 | | 9.697485 | | -6.169069315 | | 5.79E-08 | 1.31E-08 | down |
| VIT_00s0207g00050.t01 | 280 | INO80 complex subunit D-like | 0.5 | 0.108295 | | 34 | | 11.03955 | | -6.671569656 | | 1.14E-10 | 1.98E-11 | down |
| VIT_18s0001g08590.t01 | 689 | Putative uncharacterized protein | 0.5 | 0.04401 | | 42 | | 5.541922 | | -6.976424237 | | 9.61E-13 | 1.42E-13 | down |
| VIT_06s0004g00020.t01 | 1079 | NAC domain-containing protein 68-like | 0.5 | 0.028103 | | 63 | | 5.308227 | | -7.561386738 | | 6.90E-18 | 7.33E-19 | down |
| VIT_00s2301g00010.t01 | 272 | Putative uncharacterized protein | 0.5 | 0.11148 | | 63 | | 21.05727 | | -7.561386738 | | 6.90E-18 | 7.33E-19 | down |
| VIT_11s0052g01180.t01 | 1059 | probable xyloglucan endotransglucosylase/ hydrolase protein 23-like | 0.5 | 0.028633 | | 135 | | 11.58959 | | -8.660922412 | | 1.73E-33 | 1.00E-34 | down |

**Additional file 4** Gene Ontology (GO) functional annotation of transcripts

| GO_item | Cluster frquency | Genome frquency of use | P_value | q_value(BH_adjust) |
| --- | --- | --- | --- | --- |
| **biological_process** | | | | |
| cellular process | 665 out of 1038 gene | 7078 out of 10973 gene | 0.60968 | 1 |
| metabolic process | 647 out of 1038 gene | 7030 out of 10973 gene | 0.88284 | 1 |
| response to stimulus | 274 out of 1038 gene | 2416 out of 10973 gene | 0.00019 | 0.00486262 |
| biological regulation | 240 out of 1038 gene | 2537 out of 10973 gene | 0.48201 | 0.945488215 |
| Localization | 140 out of 1038 gene | 1530 out of 10973 gene | 0.65199 | 1 |
| Photosynthesis | 80 out of 1038 gene | 1290 out of 10973 gene | 0.281021 | 1 |
| regulation of signaling pathway | 90 out of 1038 gene | 3210 out of 10973 gene | 0.09201 | 0.21891821 |
| regulation of response to stimulus | 143 out of 1038 gene | 1342 out of 10973 gene | 0.80911 | 1 |
| regulation of ROS metabolic process | 12 out of 1038 gene | 1259 out of 10973 gene | 0.09201 | 1 |
| abscisic acid metabolism | 6 out of 1038 gene | 4012 out of 10973 gene | 0.03381 | 1 |
| establishment of localization | 138 out of 1038 gene | 1504 out of 10973 gene | 0.63668 | 1 |
| Signaling | 101 out of 1038 gene | 917 out of 10973 genes | 0.0432 | 0.20030097 |
| signaling process | 98 out of 1038 genes | 862 out of 10973 genes | 0.02191 | 0.124184603 |
| SOS response | 3 out of 1038 genes | 1320 out of 10973 genes | 0.43011 | 0.910301019 |
| Salinity response | 148 out of 1038 genes | 1540 out of 10973 genes | 0.30201 | 1 |
| ion homeostasis | 44 out of 1038 genes | 7210 out of 10973 genes | 0.11010 | 1 |
| ATP synthesis coupled electron transport | 14 out of 1038 genes | 2194 out of 10973 genes | 0.40019 | 1 |
| photosynthesis, light reaction | 45 out of 1038 genes | 3201 out of 10973 genes | 0.30130 | 1 |
| electron transport chain | 40 out of 1038 genes | 301 out of 10973 genes | 0.39101 | 1 |
| response to oxidative stress | 164 out of 1038 genes | 3019 out of 10973 genes | 0.59010 | 1 |
| photosynthetic electron transport chain | 15 out of 1038 genes | 3210 out of 10973 genes | 0.76021 | 0.02010310 |
| stomatal closure | 3 out of 1038 genes | 4201 out of 10973 genes | 0.19021 | 0.310310211 |
| cytochrome b6f complex assembly | 3 out of 1038 genes | 5201 out of 10973 genes | 0.40013 | 0.031030193 |
| cellular component organization | 87 out of 1038 genes | 934 out of 10973 genes | 0.53451 | 0.973580164 |
| developmental process | 77 out of 1038 genes | 1084 out of 10973 gene | 0.99764 | 1 |
| multicellular organismal process | 69 out of 1038 genes | 1105 out of 10973 gene | 0.99997 | 1 |
| cellular component biogenesis | 42 out of 1038 genes | 308 out of 10973 genes | 0.00595 | 0.043353089 |
| multi‐organism process | 41 out of 1038 genes | 404 out of 10973 genes | 0.27984 | 0.648715364 |
| cell wall organization or biogenes | 40 out of 1038 genes | 351 out of 10973 genes | 0.09079 | 0.3005265 |
| Reproduction | 33 out of 1038 genes | 564 out of 10973 genes | 0.99903 | 1 |
| reproductive process | 31 out of 1038 genes | 539 out of 10973 genes | 0.99908 | 1 |
| Growth | 11 out of 1038 genes | 147 out of 10973 genes | 0.74606 | 1 |
| rhythmic process | 8 out of 1038 genes | 56 out of 10973 genes | 0.07836 | 0.3005265 |
| Death | 5 out of 1038 genes | 120 out of 10973 genes | 0.97564 | 1 |
| immune system process | 5 out of 1038 genes | 157 out of 10973 genes | 0.99793 | 1 |
| cell killing | 4 out of 1038 genes | 11 out of 10973 genes | 0.00213 | 0.027168631 |
| **cellular_component** | | | | |
| Cell | 789 out of 1038 gene | 8436 out of 10973 gene | 0.74589 | 1 |
| cell part | 789 out of 1038 gene | 8436 out of 10973 gene | 0.64589 | 1 |
| Organelle | 554 out of 1038 gene | 5454 out of 10973 gene | 0.00591 | 0.043353089 |
| organelle part | 239 out of 1038 gene | 093 out of 10973 gene | 0.00036 | 0.006203394 |
| Photosynthetic membrane | 106 out of 1038 gene | 032 out of 10973 gene | 0.74589 | 1 |
| Thylakoid membrane | 97 out of 1038 gene | 431 out of 10973 gene | 0.64589 | 1 |
| Photosystem 1 | 17 out of 1038 gene | 7201 out of 10973 gene | 0.00591 | 0.043353089 |
| protein tyrosine/serine/threonine phosphatase complex | 8 out of 1038 gene | 4310 out of 10973 gene | 0.00036 | 0.006203394 |
| Photosystem II reaction center | 4 out of 1038 gene | 0931 out of 10973 gene | 0.74589 | 1 |
| Transcription factor TFIIF Complex | 3 out of 1038 gene | 731 out of 10973 gene | 0.64589 | 1 |
| protein kinase complex | 3 out of 1038 gene | 541 out of 10973 gene | 0.00591 | 0.043353089 |
| vacuolar hydrogen-transporting ATPase | 28 out of 1038 gene | 563 out of 10973 gene | 0.00036 | 0.006203394 |
| mitochondrial proton-transporting ATP synthase complex | 8 out of 1038 gene | 531 out of 10973 gene | 0.74589 | 1 |
| light harvesting complex | 3 out of 1038 gene | 432 out of 10973 gene | 0.64589 | 1 |
| Photosystem II | 15 out of 1038 gene | 402 out of 10973 gene | 0.00591 | 0.043353089 |
| Transcription factor Complex | 9 out of 1038 gene | 019 out of 10973 gene | 0.00036 | 0.006203394 |
| proton-transporting V-type ATPase, V1 domain | 4 out of 1038 gene | 891 out of 10973 gene | 0.74589 | 1 |
| proton-transporting V-type ATPase, V0 domain | 6 out of 1038 gene | 911 out of 10973 gene | 0.64589 | 1 |
| NADH dehydrogenase complex | 5 out of 1038 gene | 401 out of 10973 gene | 0.00591 | 0.043353089 |
| macromolecular complex | 131 out of 1038 gene | 1289 out of 10973 gene | 0.00036 | 0.006203394 |
| extracellular region | 89 out of 1038 genes | 903 out of 10973 genes | 0.31048 | 1 |
| membrane‐enclosed lumen | 50 out of 1038 genes | 444 out of 10973 genes | 0.08236 | 0.3005265 |
| Symplast | 4 out of 1038 genes | 20 out of 10973 genes | 0.03495 | 1 |
| extracellular region part | 2 out of 1038 genes | 44 out of 10973 genes | 0.79961 | 1 |
| **molecular_function** | | | | |
| Binding | 648 out of 1038 gene | 7367 out of 10973 gene | 0.99957 | 1 |
| catalytic activity | 497 out of 1038 gene | 6090 out of 10973 gene | 1 | 1 |
| ATP-dependent helicase activity | 49 out of 1038 gene | 4082 out of 10973 gene | 0.00314 | 0.344290517 |
| Photosynthesis Electron transporter | 8 out of 1038 gene | 9020 out of 10973 gene | 0.47215 | 0.401229059 |
| Catalase activity | 5 out of 1038 gene | 3291 out of 10973 gene | 1 | 0.091290021 |
| ATPase activity | 178 out of 1038 gene | 4932 out of 10973 gene | 0.12151 | 0.921290517 |
| Chlorophyll binding | 9 out of 1038 gene | 4311 out of 10973 gene | 0.97804 | 1 |
| NADH dehydrogenase (ubiquinone) activity | 26 out of 1038 gene | 7420 out of 10973 gene | 0.37215 | 0.345488215 |
| Protein tyrosine/serine/threonine phosphatase activity | 12 out of 1038 gene | 6731 out of 10973 gene | 0.17205 | 1 |
| Phosphatase activity | 113 out of 1038 gene | 9402 out of 10973 gene | 0.10018 | 1 |
| Sodium transporter activity | 7 out of 1038 gene | 492 out of 10973 gene | 0.12151 | 0.344290517 |
| Heat shock protein binding | 18 out of 1038 gene | 3920 out of 10973 gene | 0.97804 | 1 |
| Superoxide dismutase activity | 8 out of 1038 gene | 4301 out of 10973 gene | 0.32215 | 0.945488215 |
| Transcription factor binding | 8 out of 1038 gene | 5030 out of 10973 gene | 0.12151 | 0.344290517 |
| ABA binding | 4 out of 1038 gene | 6013 out of 10973 gene | 0.97804 | 1 |
| Hsp90 protein binding | 4 out of 1038 gene | 8210 out of 10973 gene | 0.47215 | 0.945488215 |
| Potassium transporter activity | 16 out of 1038 gene | 340 out of 10973 gene | 0.99615 | 0.201100103 |
| transcription regulator activity | 74 out of 1038 genes | 695 out of 10973 genes | 0.12151 | 0.344290517 |
| transporter activity | 56 out of 1038 genes | 759 out of 10973 genes | 0.97804 | 1 |
| structural molecule activity | 34 out of 1038 genes | 362 out of 10973 genes | 0.47215 | 0.945488215 |
| molecular transducer activity | 24 out of 1038 genes | 638 out of 10973 genes | 1 | 1 |
| electron carrier activity | 23 out of 1038 genes | 346 out of 10973 genes | 0.96229 | 1 |
| antioxidant activity | 17 out of 1038 genes | 138 out of 10973 genes | 0.10018 | 0.3005265 |
| enzyme regulator activity | 11 out of 1038 genes | 131 out of 10973 genes | 0.59102 | 1 |
| protein tag | 2 out of 1038 genes | 5 out of 10973 genes | 0.00729 | 0.046492072 |
| translation regulator activity | 2 out of 1038 genes | 4 out of 10973 genes | 0.00314 | 0.032008549 |
| nutrient reservoir activity | 1 out of 1038 genes | 78 out of 10973 genes | 0.99615 | 1 |
| proteasome regulator activity | 1 out of 1038 genes | 1 out of 10973 genes | 0 | 0 |

**Additional file 5.** KEGG pathway mapping

| KEGG_pathway | Cluster frquency | Genome frquency of use | P_value | q_value (BH_adjust) |
| --- | --- | --- | --- | --- |
| Signal transduction | 79 out of 453 genes | 543 out of 4132 genes | 0.0021879 | 0.035006528 |
| Folding, sorting and degradation | 65 out of 453 genes | 465 out of 4132 genes | 0.0129091 | 0.137697493 |
| Carbohydrate metabolism | 63 out of 453 genes | 594 out of 4132 genes | 0.5861103 | 0.9989584 |
| Energy metabolism | 60 out of 453 genes | 360 out of 4132 genes | 0.0002179 | 0.006974064 |
| Translation | 59 out of 453 genes | 592 out of 4132 genes | 0.7770667 | 0.9989584 |
| Overview | 53 out of 453 genes | 395 out of 4132 genes | 0.0450138 | 0.288088448 |
| Amino acid metabolism | 48 out of 453 genes | 444 out of 4132 genes | 0.5047592 | 0.9989584 |
| Lipid metabolism | 29 out of 453 genes | 351 out of 4132 genes | 0.9493836 | 0.9989584 |
| Endocrine system | 29 out of 453 genes | 236 out of 4132 genes | 0.2153073 | 0.68898336 |
| Transcription | 26 out of 453 genes | 235 out of 4132 genes | 0.4276062 | 0.91222656 |
| Transport and catabolism | 25 out of 453 genes | 308 out of 4132 genes | 0.9455067 | 0.9989584 |
| Environmental adaptation | 23 out of 453 genes | 146 out of 4132 genes | 0.0262172 | 0.20973752 |
| Metabolism of cofactors and vitamins | 22 out of 453 genes | 256 out of 4132 genes | 0.8767461 | 0.9989584 |
| Cell growth and death | 21 out of 453 genes | 211 out of 4132 genes | 0.635296 | 0.9989584 |
| Metabolism of other amino acids | 21 out of 453 genes | 187 out of 4132 genes | 0.3951688 | 0.903242971 |
| Nucleotide metabolism | 19 out of 453 genes | 165 out of 4132 genes | 0.3500897 | 0.903242971 |
| Biosynthesis of other secondary metabolit | 19 out of 453 genes | 204 out of 4132 genes | 0.739849 | 0.9989584 |
| Xenobiotics biodegradation and metaboli | 17 out of 453 genes | 181 out of 4132 genes | 0.708878 | 0.9989584 |
| Immune system | 16 out of 453 genes | 160 out of 4132 genes | 0.5945755 | 0.9989584 |
| Nervous system | 13 out of 453 genes | 177 out of 4132 genes | 0.9321202 | 0.9989584 |
| Glycan biosynthesis and metabolism | 13 out of 453 genes | 114 out of 4132 genes | 0.3675651 | 0.903242971 |
| Metabolism of terpenoids and polyketide | 10 out of 453 genes | 182 out of 4132 genes | 0.992971 | 0.9989584 |
| Cell motility | 8 out of 453 genes | 57 out of 4132 genes | 0.1663711 | 0.6654844 |
| Circulatory system | 7 out of 453 genes | 55 out of 4132 genes | 0.2509037 | 0.729901673 |
| Development | 6 out of 453 genes | 34 out of 4132 genes | 0.0715335 | 0.38151216 |
| Excretory system | 5 out of 453 genes | 64 out of 4132 genes | 0.7182747 | 0.9989584 |
| Replication and repair | 5 out of 453 genes | 142 out of 4132 genes | 0.9989584 | 0.9989584 |
| Digestive system | 5 out of 453 genes | 84 out of 4132 genes | 0.9117114 | 0.9989584 |
| Sensory system | 4 out of 453 genes | 25 out of 4132 genes | 0.1310776 | 0.599211886 |
| Cellular commiunity | 4 out of 453 genes | 71 out of 4132 genes | 0.9028167 | 0.9989584 |

**Additional file 6** Differently-expressed genes related to ROS producing and scavenging system

| #Gene | Length | Function | read_A | RPKM_A | read_B | RPKM_B | log2 | q-value | p_value | Result |
| --- | --- | --- | --- | --- | --- | --- | --- | --- | --- | --- |
| **SOD** | | | | | | | | | | |
| VIT_10s0042g00100.t01 | 1255 | superoxide dismutase [Fe] | 256 | 12.37066 | 343 | 24.84739 | -1.006171581 | 8.74E-17 | 9.81E-18 | down |
| **POD** | | | | | | | | | | |
| VIT_13s0067g02360.t01 | 1022 | peroxidase 4 | 115 | 6.824073 | 6 | 0.533741 | 3.676420736 | 1.11E-19 | 1.06E-20 | up |
| VIT_08s0040g02200.t01 | 1024 | peroxidase 21 | 171 | 10.12728 | 10 | 0.887831 | 3.511817605 | 7.29E-28 | 5.01E-29 | up |
| VIT_06s0004g07770.t01 | 1205 | Peroxidase 4 | 789 | 39.70879 | 166 | 12.52424 | 1.664735244 | 5.79E-54 | 2.04E-55 | up |
| VIT_16s0098g00820.t01 | 2398 | peroxidase 5 | 239 | 6.044292 | 59 | 2.236831 | 1.434116944 | 7.47E-14 | 1.01E-14 | up |
| VIT_12s0055g00810.t01 | 1199 | peroxidase 43 | 1091 | 55.18261 | 291 | 22.06501 | 1.322453229 | 4.91E-53 | 1.76E-54 | up |
| VIT_07s0191g00050.t01 | 1204 | peroxidase 17 | 146 | 7.353991 | 40 | 3.020396 | 1.283789649 | 1.18E-07 | 2.75E-08 | up |
| VIT_12s0059g02420.t01 | 2148 | peroxidase 3 | 566 | 15.98008 | 169 | 7.152911 | 1.159671992 | 6.85E-23 | 5.62E-24 | up |
| VIT_18s0072g00160.t01 | 1154 | peroxidase 12 | 375 | 19.70707 | 123 | 9.690132 | 1.024125465 | 7.37E-13 | 1.07E-13 | up |
| **CAT** | | | | | | | | | | |
| VIT_04s0044g00020.t01 | 1763 | catalase isozyme 1 | 25613 | 881.0587 | 7656 | 394.8026 | 1.158106693 | 0 | 0 | up |
| VIT_00s0698g00010.t01 | 1763 | catalase isozyme 1 | 18048 | 620.8311 | 5743 | 296.1535 | 1.067855691 | 0 | 0 | up |
| **GSH-AsA cycle** | | | | | | | | | | |
| VIT_08s0007g03240.t01 | 1161 | monodehydroascorbate reductase（MDAR） | 2646 | 138.2147 | 804 | 62.95848 | 1.134438841 | 2.12E-100 | ######## | up |
| VIT_08s0040g03150.t01 | 1071 | cytosolic ascorbate peroxidase (APX) | 1384 | 78.36882 | 338 | 28.69179 | 1.449641977 | 5.00E-77 | 1.28E-78 | up |
| VIT_12s0028g03010.t01 | 661 | glutaredoxin-C9（GRX） | 1287 | 118.0793 | 2725 | 374.7964 | -1.666350991 | 3.64E-277 | ######## | down |
| VIT_17s0000g08670.t01 | 653 | glutaredoxin-C1（GRX） | 1141 | 105.9666 | 317 | 44.13432 | 1.263637232 | 1.05E-51 | 3.86E-53 | up |
| VIT_07s0141g00390.t01 | 1983 | glutathione reductase（GR） | 402 | 12.29419 | 847 | 38.83212 | -1.659273283 | 5.13E-86 | 1.18E-87 | down |
| VIT_05s0051g00870.t01 | 2217 | glutathione reductase（GR） | 277 | 7.577235 | 395 | 16.19801 | -1.096073492 | 6.47E-22 | 5.58E-23 | down |
| **GST** | | | | | | | | | | |
| VIT_05s0049g01070.t01 | 752 | glutathione S-transferase | 135 | 10.88711 | 3 | 0.362689 | 4.907746282 | 1.59E-26 | 1.14E-27 | up |
| VIT_05s0049g01100.t01 | 823 | glutathione S-transferase | 197 | 14.51654 | 6 | 0.662799 | 4.452982504 | 6.00E-37 | 3.12E-38 | up |
| VIT_06s0004g05670.t01 | 1329 | glutathione S-transferase | 168 | 7.666215 | 22 | 1.504971 | 2.34877899 | 1.19E-18 | 1.21E-19 | up |
| VIT_05s0049g01090.t01 | 795 | glutathione transferase GST 23 | 92 | 7.018065 | 13 | 1.486643 | 2.239015423 | 3.87E-10 | 7.05E-11 | up |
| VIT_05s0049g01120.t01 | 931 | glutathione transferase GST 23 | 91 | 5.92773 | 13 | 1.269475 | 2.223248107 | 5.80E-10 | 1.07E-10 | up |
| VIT_07s0005g04890.t01 | 999 | glutathione transferase GST 23 | 482 | 29.26026 | 89 | 8.099438 | 1.853049091 | 1.66E-38 | 8.24E-40 | up |
| VIT_17s0000g02930.t01 | 886 | glutathione S-transferase | 463 | 31.69159 | 107 | 10.97945 | 1.529294582 | 2.02E-28 | 1.36E-29 | up |
| VIT_19s0027g00460.t01 | 822 | glutathione S-transferase | 569 | 41.97949 | 169 | 18.69155 | 1.167298591 | 3.07E-23 | 2.48E-24 | up |
| VIT_19s0093g00150.t01 | 706 | glutathione S-transferase | 449 | 38.569 | 641 | 82.54366 | -1.097715726 | 4.41E-35 | 2.42E-36 | down |
| VIT_19s0015g02590.t01 | 880 | glutathione S-transferase | 43 | 2.963347 | 63 | 6.50861 | -1.135121983 | 0.000141086 | 5.02E-05 | down |
| VIT_14s0006g00630.t01 | 906 | glutathione S-transferase | 472 | 31.59443 | 696 | 69.84116 | -1.144407261 | 1.35E-40 | 6.36E-42 | down |
| VIT_08s0040g03040.t01 | 961 | glutathione S-transferase | 81 | 5.111617 | 154 | 14.56893 | -1.511043352 | 2.03E-14 | 2.64E-15 | down |
| VIT_04s0079g00690.t01 | 831 | glutathione S-transferase | 660 | 48.16589 | 1260 | 137.8478 | -1.516992619 | 3.62E-112 | ######## | down |
| VIT_19s0093g00160.t01 | 892 | glutathione S-transferase | 26 | 1.767686 | 51 | 5.197993 | -1.556092438 | 1.11E-05 | 3.33E-06 | down |
| VIT_08s0040g03100.t01 | 768 | glutathione S-transferase | 68 | 5.36963 | 162 | 19.17715 | -1.836493976 | 1.26E-19 | 1.21E-20 | down |
| VIT_19s0093g00110.t01 | 674 | glutathione S-transferase | 12 | 1.079737 | 77 | 10.38631 | -3.265930855 | 4.60E-18 | 4.85E-19 | down |
| **TRX** | | | | | | | | | | |
| VIT_08s0040g03130.t01 | 946 | type II peroxiredoxin 2 | 155 | 9.936588 | 237 | 22.77653 | -1.196725658 | 1.93E-15 | 2.34E-16 | down |
| VIT_00s0532g00030.t01 | 533 | thioredoxin h (TRX) | 925 | 105.2474 | 169 | 28.82636 | 1.868323305 | 5.47E-74 | 1.45E-75 | up |
| VIT_05s0020g04250.t01 | 662 | thioredoxin protein 4A | 3835 | 351.321 | 787 | 108.0804 | 1.700684222 | 7.63E-268 | ######## | up |
| VIT_08s0007g07620.t01 | 1448 | thioredoxin | 2646 | 110.82 | 631 | 39.61787 | 1.483994337 | 6.87E-152 | ######## | up |
| VIT_04s0023g02700.t01 | 556 | thioredoxin H-type 2 | 5279 | 575.8025 | 1740 | 284.5148 | 1.017070545 | 2.63E-167 | ######## | up |
| VIT_01s0011g01380.t01 | 874 | thioredoxin | 72 | 4.995946 | 101 | 10.50607 | -1.072393296 | 3.37E-06 | 9.42E-07 | down |
| VIT_01s0011g01370.t01 | 672 | thioredoxin | 69 | 6.226966 | 97 | 13.12299 | -1.0754952 | 5.10E-06 | 1.46E-06 | down |
| VIT_04s0023g02390.t01 | 1233 | thioredoxin | 56 | 2.754366 | 93 | 6.857254 | -1.315910704 | 1.33E-07 | 3.11E-08 | down |
| VIT_01s0026g01460.t01 | 609 | thioredoxin H2 | 66 | 6.57239 | 172 | 25.67684 | -1.96597745 | 1.13E-22 | 9.38E-24 | down |
| **AOX** | | | | | | | | | | |
| VIT_02s0033g01380.t01 | 1185 | alternative oxidase 3 | 76 | 3.889484 | 139 | 10.66416 | -1.455120374 | 1.84E-12 | 2.78E-13 | down |
| **PPO** | | | | | | | | | | |
| VIT_00s0480g00080.t01 | 267 | polyphenol oxidase | 236 | 53.60403 | 334 | 113.7275 | -1.085168058 | 2.12E-18 | 2.19E-19 | down |

**Additional file 7** Differently-expressed genes related to heat shock protein (HSP) and pathogenesis-related proteins (PR)

| #Gene | Length | Function | read_A | RPKM_A | read_B | RPKM_B | log2 | q-value | p_value | Result |
| --- | --- | --- | --- | --- | --- | --- | --- | --- | --- | --- |
| **Disease related proteins** | | | | | | | | | | |
| VIT_00s0144g00040.t01 | 592 | disease resistance protein RGA2 | 49 | 5.019623 | 1 | 0.153571 | 5.03060303 | 2.45E-10 | 4.39E-11 | up |
| VIT_13s0064g00350.t01 | 135 | disease resistance RPP13 protein 1 | 16 | 7.187584 | 0.5 | 0.336718 | 4.415893185 | 0.000590802 | 0.00023578 | up |
| VIT_02s0025g00730.t01 | 599 | disease resistance response protein | 89 | 9.010728 | 7 | 1.062433 | 3.084271694 | 1.70E-13 | 2.35E-14 | up |
| VIT_12s0034g01690.t01 | 288 | disease resistance RPP13 protein 1 | 36 | 7.580655 | 3 | 0.94702 | 3.000855686 | 5.77E-06 | 1.67E-06 | up |
| VIT_12s0034g01710.t01 | 389 | disease resistance RPP13 protein 1 | 33 | 5.144712 | 5 | 1.168559 | 2.13835921 | 0.000381376 | 0.0001478 | up |
| VIT_02s0025g00740.t01 | 775 | disease resistance response protein 206 | 279 | 21.83229 | 44 | 5.161564 | 2.080582879 | 2.95E-26 | 2.13E-27 | up |
| VIT_18s0117g00070.t01 | 949 | disease resistance protein | 719 | 45.94723 | 158 | 15.13635 | 1.601960398 | 1.19E-46 | 4.87E-48 | up |
| VIT_12s0034g01750.t01 | 2968 | disease resistance RPP13 protein 1 | 265 | 5.414753 | 64 | 1.960408 | 1.465741735 | 1.02E-15 | 1.21E-16 | up |
| VIT_06s0004g00980.t01 | 778 | disease resistance response protein 206 | 571 | 44.50955 | 188 | 21.96892 | 1.018651269 | 6.84E-19 | 6.87E-20 | up |
| VIT_04s0008g07260.t01 | 1133 | protein enhanced disease resistance 2 | 105 | 5.620256 | 150 | 12.03626 | -1.098679987 | 5.42271E-09 | 1.09924E-09 | down |
| VIT_14s0030g01000.t01 | 568 | disease resistance protein | 39 | 4.164022 | 58 | 9.283463 | -1.156685591 | 0.000212591 | 7.82346E-05 | down |
| VIT_15s0024g00400.t01 | 3465 | disease resistance protein RGA4 | 98 | 1.715219 | 205 | 5.378745 | -1.64887707 | 2.78E-21 | 2.48E-22 | down |
| VIT_13s0064g01840.t01 | 132 | disease resistance protein | 3 | 1.378301 | 18 | 12.39735 | -3.169069315 | 6.28E-05 | 2.10E-05 | down |
| **pathogenesis-related proteins** | | | | | | | | | | |
| VIT_03s0088g00890.t01 | 489 | pathogenesis-related protein 1 (PR-1) | 41 | 5.084775 | 0.5 | 0.092959 | 5.77344519 | 5.80E-09 | 1.18E-09 | up |
| VIT_03s0088g00910.t01 | 1005 | pathogenesis-related protein 1 (PR-1) | 259 | 15.62897 | 25 | 2.26154 | 2.788845283 | 5.41E-34 | 3.07E-35 | up |
| VIT_11s0052g01650.t01 | 826 | pathogenesis-related protein 1 (PR-1) | 5486 | 402.7842 | 1477 | 162.5664 | 1.308977981 | 5.09E-260 | 4.50E-262 | up |
| VIT_03s0088g00810.t01 | 683 | pathogenesis-related protein 1 (PR-1) | 3330 | 295.6788 | 1080 | 143.7585 | 1.04038405 | 9.7493E-110 | 1.8036E-111 | up |
| VIT_08s0007g06060.t01 | 1186 | beta 1-3 glucanase | 80 | 4.090741 | 247 | 18.93401 | -2.210545951 | 3.21E-37 | 1.65E-38 | down |
| VIT_11s0149g00350.t01 | 1101 | chitotriosidase-1 | 12 | 0.660984 | 76 | 6.27562 | -3.247071827 | 9.35E-18 | 9.98E-19 | down |
| VIT_11s0206g00030.t01 | 1136 | chitotriosidase-1 | 4 | 0.21354 | 71 | 5.68212 | -4.733853934 | 7.14E-21 | 6.44E-22 | down |
| VIT_08s0007g00760.t01 | 1008 | thaumatin protein | 151 | 9.084753 | 38 | 3.42731 | 1.406370411 | 6.62E-09 | 1.36E-09 | up |
| VIT_02s0025g04270.t01 | 823 | thaumatin protein | 1855 | 136.6913 | 2618 | 289.2013 | -1.081152725 | 1.51E-137 | 2.21E-139 | down |
| VIT_02s0025g04250.t01 | 713 | thaumatin protein | 279 | 23.73075 | 465 | 59.29169 | -1.321072409 | 3.86E-34 | 2.18E-35 | down |
| VIT_02s0025g04230.t01 | 701 | thaumatin protein | 741 | 64.10574 | 1260 | 163.4116 | -1.349985101 | 3.15E-94 | 6.72E-96 | down |
| VIT_02s0025g04260.t01 | 642 | thaumatin protein | 118 | 11.14663 | 207 | 29.31337 | -1.394950723 | 5.30E-17 | 5.88E-18 | down |
| VIT_15s0048g00020.t01 | 573 | thaumatin protein | 9 | 0.952543 | 33 | 5.23588 | -2.458575933 | 1.62E-06 | 4.34E-07 | down |
| VIT_05s0020g03740.t01 | 368 | non-specific lipid-transfer protein protein | 33 | 5.438296 | 0.5 | 0.123524 | 5.460287305 | 2.11E-07 | 5.07E-08 | up |
| VIT_14s0006g02550.t01 | 556 | non-specific lipid-transfer protein protein | 7521 | 820.3468 | 365 | 59.6827 | 3.780849313 | 0 | 0 | up |
| VIT_14s0006g02570.t01 | 1460 | non-specific lipid-transfer protein protein | 10030 | 416.6245 | 686 | 42.71709 | 3.285862405 | 0 | 0 | up |
| VIT_13s0019g02690.t01 | 603 | non-specific lipid-transfer protein protein | 150 | 15.08588 | 13 | 1.960002 | 2.944272158 | 3.81E-21 | 3.40E-22 | up |
| VIT_14s0006g02530.t01 | 602 | non-specific lipid-transfer protein protein | 6041 | 608.5679 | 944 | 142.5627 | 2.093821807 | 0 | 0 | up |
| VIT_08s0058g01210.t01 | 652 | non-specific lipid-transfer protein protein | 176044 | 16374.59 | 33364 | 4652.227 | 1.815465074 | 0 | 0 | up |
| VIT_00s0333g00050.t01 | 523 | non-specific lipid-transfer protein protein | 12672 | 1469.4 | 3194 | 555.2181 | 1.404101208 | 0 | 0 | up |
| VIT_16s0013g00070.t01 | 562 | non-specific lipid-transfer protein protein | 2 | 0.215819 | 48 | 7.76489 | -5.169069315 | 8.98E-15 | 1.13E-15 | down |
| VIT_17s0000g05360.t01 | 799 | germin protein subfamily T | 17 | 1.290324 | 50 | 5.689231 | -2.140500163 | 4.68113E-08 | 1.0485E-08 | down |
| VIT_17s0000g07770.t01 | 209 | snakin-2 | 18 | 5.223035 | 0.5 | 0.217497 | 4.585818187 | 0.00022667 | 8.39E-05 | up |
| VIT_18s0001g09460.t01 | 662 | snakin-1 | 132 | 12.0924 | 36 | 4.943959 | 1.290362303 | 4.51E-07 | 1.13E-07 | up |
| **LMW Heat shock proteins** | | | | | | | | | | |
| VIT_16s0098g01060.t01 | 854 | small heat shock protein, chloroplastic | 324 | 23.00826 | 16 | 1.703305 | 3.755743188 | 1.24E-54 | 4.31E-56 | up |
| VIT_13s0019g00860.t01 | 809 | 15.7 kDa heat shock protein, peroxisomal | 262 | 19.64036 | 17 | 1.910428 | 3.361853346 | 8.73E-41 | 4.08E-42 | up |
| VIT_12s0035g01910.t01 | 1183 | 22.0 kDa class IV heat shock protein | 606 | 31.06595 | 46 | 3.535114 | 3.135505213 | 7.19E-88 | 1.63E-89 | up |
| VIT_06s0004g05770.t01 | 670 | 18.2 kDa class I heat shock protein | 74 | 6.698131 | 10 | 1.356924 | 2.303418456 | 1.28E-08 | 2.71E-09 | up |
| VIT_04s0008g01590.t01 | 716 | 17.9 kDa class II heat shock protein | 667 | 56.49493 | 91 | 11.5547 | 2.289641496 | 2.77E-69 | 7.75E-71 | up |
| VIT_13s0019g02930.t01 | 756 | 18.2 kDa class I heat shock protein | 495 | 39.70819 | 72 | 8.658468 | 2.197252899 | 4.08E-49 | 1.59E-50 | up |
| VIT_18s0089g01270.t01 | 757 | 22.0 kDa class IV heat shock protein | 71 | 5.687995 | 13 | 1.561269 | 1.865200587 | 1.35E-06 | 3.57E-07 | up |
| VIT_04s0008g01570.t01 | 552 | 17.9 kDa class II heat shock protein | 98 | 10.76673 | 22 | 3.623381 | 1.571171411 | 4.59E-07 | 1.15E-07 | up |
| VIT_13s0019g03000.t01 | 752 | 18.2 kDa class I heat shock protein | 291 | 23.46777 | 79 | 9.550797 | 1.29698778 | 2.02E-14 | 2.62E-15 | up |
| VIT_04s0008g01490.t01 | 524 | small heat shock protein 17.3 kDa | 691 | 79.97301 | 191 | 33.13847 | 1.271006258 | 6.25E-32 | 3.76E-33 | up |
| VIT_00s0992g00020.t01 | 737 | 15.4 kDa class V heat shock protein | 483 | 39.74444 | 134 | 16.5298 | 1.265683374 | 1.89E-22 | 1.59E-23 | up |
| VIT_09s0002g00630.t01 | 219 | 17.4 kDa class III heat shock protein | 492 | 136.2441 | 142 | 58.94875 | 1.208660571 | 2.54E-21 | 2.25E-22 | up |
| VIT_00s0707g00010.t01 | 688 | 15.4 kDa class V heat shock protein | 507 | 44.6906 | 157 | 20.74634 | 1.107114374 | 3.40E-19 | 3.33E-20 | up |
| VIT_04s0008g01580.t01 | 681 | 17.9 kDa class II heat shock protein | 909 | 80.94937 | 282 | 37.64717 | 1.104478317 | 1.46E-33 | 8.46E-35 | up |
| VIT_13s0019g03170.t01 | 742 | 18.2 kDa class I heat shock protein | 8059 | 658.6792 | 2646 | 324.2025 | 1.022680957 | 1.62E-257 | 1.45E-259 | up |
| VIT_04s0008g01510.t01 | 976 | 17.9 kDa class II heat shock protein | 124 | 7.704928 | 41 | 3.81913 | 1.012537491 | 6.84E-05 | 2.30E-05 | up |
| VIT_13s0019g02840.t01 | 628 | 18.2 kDa class I heat shock protein | 56 | 5.407856 | 79 | 11.43662 | -1.080532641 | 3.94E-05 | 1.28E-05 | down |
| **HMW Heat shock proteins** | | | | | | | | | | |
| VIT_18s0041g01230.t01 | 2429 | stromal 70 kDa heat shock-related protein | 323 | 8.064393 | 73 | 2.732283 | 1.561458981 | 1.06E-20 | 9.65E-22 | up |
| VIT_00s0415g00030.t01 | 2305 | heat shock 70 kDa protein, mitochondrial | 412 | 10.83984 | 788 | 31.08033 | -1.519658107 | 1.43E-70 | 3.91E-72 | down |
| VIT_13s0019g01430.t01 | 2052 | heat shock 70kDa protein 1/8 | 8423 | 248.9351 | 11908 | 527.5843 | -1.08363187 | 0 | 0 | down |
| VIT_00s0194g00030.t01 | 2147 | heat shock cognate protein 80 | 2334 | 65.92733 | 3511 | 148.672 | -1.17318425 | 3.96E-210 | 4.07E-212 | down |
| **HSF** | | | | | | | | | | |
| VIT_07s0005g00120.t01 | 650 | heat shock factor-binding protein 1 | 226 | 21.08588 | 57 | 7.972451 | 1.403182134 | 9.13E-13 | 1.34E-13 | up |
| VIT_05s0020g01500.t01 | 232 | heat shock factor-binding protein 1 | 436 | 113.9712 | 144 | 56.42933 | 1.014152509 | 1.47E-14 | 1.88E-15 | up |
| VIT_11s0016g03940.t01 | 1113 | heat stress transcription factor C-1 | 588 | 32.03899 | 196 | 16.01 | 1.000855686 | 6.77E-19 | 6.79E-20 | up |
| VIT_04s0008g01110.t01 | 1415 | heat shock factor protein HSF30 | 149 | 6.385965 | 202 | 12.97852 | -1.023149777 | 1.43E-10 | 2.51E-11 | down |
| VIT_07s0031g00670.t01 | 1671 | heat shock factor protein HSF24 | 151 | 5.48021 | 230 | 12.51359 | -1.191192126 | 6.52E-15 | 8.16E-16 | down |
| VIT_16s0039g01840.t01 | 1841 | heat stress transcription factor A-1 | 134 | 4.414156 | 278 | 13.72845 | -1.636958697 | 3.03E-28 | 2.06E-29 | down |
| **other Heat shock proteins** | | | | | | | | | | |
| VIT_15s0046g01560.t01 | 4073 | BAG family molecular chaperone regulator 6 | 8654 | 128.8544 | 732 | 16.33906 | 2.979344753 | 0 | 0 | up |
| VIT_03s0110g00290.t01 | 822 | chaperone protein dnaJ 11, chloroplastic | 79 | 5.828435 | 16 | 1.769614 | 1.719673934 | 1.44E-06 | 3.83E-07 | up |
| VIT_18s0122g00070.t01 | 439 | chaperone protein dnaJ 10 | 233 | 32.18756 | 56 | 11.59722 | 1.472724408 | 4.87E-14 | 6.51E-15 | up |
| VIT_14s0060g01490.t01 | 758 | chaperone protein DnaJ | 11036 | 882.9563 | 3020 | 362.2164 | 1.285490093 | 0 | 0 | up |
| VIT_03s0038g02110.t01 | 575 | chaperone protein dnaJ 11, chloroplastic | 790 | 83.32128 | 230 | 36.36557 | 1.196111978 | 3.78E-33 | 2.21E-34 | up |
| VIT_09s0018g00620.t01 | 735 | chaperone protein dnaJ 8, chloroplasti | 929 | 76.65228 | 277 | 34.2628 | 1.161685806 | 4.23E-37 | 2.19E-38 | up |
| VIT_05s0029g00500.t01 | 951 | chaperone protein dnaJ 20, chloroplastic | 640 | 40.81278 | 212 | 20.26682 | 1.009900826 | 9.05E-21 | 8.22E-22 | up |
| VIT_00s0324g00040.t01 | 2037 | chaperone protein DnaJ | 689 | 20.5128 | 1006 | 44.89907 | -1.130161232 | 3.59E-57 | 1.20E-58 | down |
| VIT_08s0032g00960.t01 | 1424 | iron-sulfur cluster co-chaperone protein HscB | 56 | 2.384925 | 82 | 5.235212 | -1.134303897 | 1.25E-05 | 3.78E-06 | down |
| VIT_01s0011g04480.t01 | 764 | histone chaperone ASF1B | 33 | 2.619493 | 63 | 7.496828 | -1.516992619 | 1.48E-06 | 3.94E-07 | down |
| VIT_08s0007g07960.t01 | 1061 | chaperone protein dnaJ 6 | 34 | 1.943391 | 67 | 5.74103 | -1.562733164 | 3.64E-07 | 9.01E-08 | down |
| VIT_15s0048g02990.t01 | 1847 | probable mitochondrial chaperone bcs1 | 239 | 7.847435 | 473 | 23.28223 | -1.56893638 | 1.12093E-44 | 4.83168E-46 | down |

**Additional file 8** Differently-expressed genes related to plant hormones

| #Gene | Length | Function | read_A | RPKM_A | read_B | RPKM_B | log2 | q-value | p_value | Result |
| --- | --- | --- | --- | --- | --- | --- | --- | --- | --- | --- |
| **auxin** | | | | | | | | | | |
| VIT_04s0023g00530.t01 | 370 | auxin-induced protein 15A | 69 | 11.30952 | 0.5 | 0.122857 | 6.524417642 | 3.71E-14 | 4.90E-15 | up |
| VIT_03s0038g01100.t01 | 269 | auxin-induced protein 15A | 51 | 11.4978 | 0.5 | 0.168985 | 6.088318527 | 7.31E-11 | 1.25E-11 | up |
| VIT_03s0038g01090.t01 | 404 | auxin-induced protein X15 | 49 | 7.355487 | 0.5 | 0.112517 | 6.03060303 | 1.73E-10 | 3.07E-11 | up |
| VIT_03s0038g01130.t01 | 975 | auxin-induced protein 10A5 | 109 | 6.779827 | 2 | 0.18649 | 5.18407751 | 6.96E-22 | 6.01E-23 | up |
| VIT_04s0023g00520.t01 | 303 | auxin-induced protein 15A | 26 | 5.203882 | 0.5 | 0.150023 | 5.116332904 | 5.25E-06 | 1.51E-06 | up |
| VIT_03s0038g03450.t01 | 380 | auxin-induced protein 6B | 132 | 21.06624 | 6 | 1.435483 | 3.875324804 | 3.03E-23 | 2.45E-24 | up |
| VIT_03s0038g00950.t01 | 683 | auxin-induced protein 15A | 714 | 63.3978 | 35 | 4.658839 | 3.766390432 | 8.21E-120 | 1.40E-121 | up |
| VIT_03s0038g01120.t01 | 267 | auxin-induced protein 15A | 53 | 12.03819 | 3 | 1.021505 | 3.558851139 | 2.01E-09 | 3.92E-10 | up |
| VIT_10s0003g00090.t01 | 724 | auxin-repressed 12.5 kDa protein | 2549 | 213.5148 | 306 | 38.42494 | 2.474221001 | 3.94E-288 | 3.10E-290 | up |
| VIT_04s0023g00580.t01 | 491 | auxin-induced protein 15A | 761 | 93.99394 | 121 | 22.40445 | 2.068782592 | 1.16E-69 | 3.22E-71 | up |
| VIT_03s0038g01110.t01 | 541 | auxin-induced protein X15 | 72 | 8.071085 | 12 | 2.016575 | 2.000855686 | 3.19E-07 | 7.82E-08 | up |
| VIT_03s0038g03760.t01 | 474 | auxin-induced protein 6B | 75 | 9.595765 | 13 | 2.49342 | 1.944272158 | 3.05E-07 | 7.47E-08 | up |
| VIT_15s0048g02860.t01 | 687 | auxin-induced protein 10A5 | 1625 | 143.4476 | 392 | 51.87519 | 1.467407344 | 5.65E-92 | 1.23E-93 | up |
| VIT_09s0002g00700.t01 | 934 | auxin-repressed 12.5 kDa protein | 779 | 50.58098 | 225 | 21.9011 | 1.207591512 | 3.48E-33 | 2.03E-34 | up |
| VIT_13s0019g04380.t01 | 2359 | auxin response factor 18 | 163 | 4.190409 | 218 | 8.40154 | -1.003562985 | 5.29E-11 | 8.95E-12 | down |
| VIT_11s0016g04490.t01 | 1617 | auxin-responsive protein IAA7 | 89 | 3.337926 | 161 | 9.052035 | -1.439290262 | 4.74E-14 | 6.33E-15 | down |
| VIT_09s0002g03410.t01 | 1999 | auxin-responsive protein IAA18 | 62 | 1.880943 | 123 | 5.594003 | -1.57242501 | 2.27E-12 | 3.45E-13 | down |
| VIT_00s1203g00010.t01 | 696 | auxin response factor 6 | 29 | 2.526885 | 59 | 7.706783 | -1.608768869 | 1.14E-06 | 3.00E-07 | down |
| VIT_18s0001g03170.t01 | 1365 | auxin-induced protein 5NG4 | 40 | 1.77715 | 83 | 5.528099 | -1.637218151 | 3.79E-09 | 7.58E-10 | down |
| VIT_07s0005g04380.t01 | 1221 | auxin-responsive protein IAA11 | 80 | 3.97348 | 166 | 12.36012 | -1.637218151 | 2.97E-17 | 3.25E-18 | down |
| VIT_03s0038g01080.t01 | 285 | indole-3-acetic acid-induced protein ARG7 | 58 | 12.34184 | 1 | 0.318996 | 5.273874181 | 3.72E-12 | 5.75E-13 | up |
| VIT_03s0038g03460.t01 | 320 | indole-3-acetic acid-induced protein ARG7 | 27 | 5.116942 | 1 | 0.284106 | 4.170780688 | 8.49E-06 | 2.51E-06 | up |
| VIT_18s0001g13980.t01 | 448 | indole-3-acetic acid-induced protein ARG7 | 290 | 39.25696 | 14 | 2.84106 | 3.788447353 | 2.82E-49 | 1.09E-50 | up |
| **ethylene** | | | | | | | | | | |
| VIT_03s0063g01340.t01 | 1173 | 1-aminocyclopropane-1-carboxylate oxidase homolog 5（ACO） | 227 | 11.73612 | 51 | 3.952779 | 1.570016331 | 5.93E-15 | 7.40E-16 | up |
| VIT_08s0007g03040.t01 | 1237 | 1-aminocyclopropane-1-carboxylate oxidase homolog 11 | 1248 | 61.18452 | 303 | 22.26913 | 1.458121421 | 3.98E-70 | 1.10E-71 | up |
| VIT_05s0049g00410.t01 | 1285 | 1-aminocyclopropane-1-carboxylate oxidase homolog 1 | 154 | 7.26799 | 48 | 3.396006 | 1.097717225 | 1.74E-06 | 4.66E-07 | up |
| VIT_05s0049g00510.t01 | 1264 | ethylene-responsive transcription factor 1B | 221 | 10.60332 | 8 | 0.575405 | 4.203795745 | 3.43E-40 | 1.63E-41 | up |
| VIT_18s0001g10150.t01 | 780 | ethylene-responsive transcription factor ERF010 | 534 | 41.51866 | 56 | 6.527153 | 2.669234195 | 4.02E-66 | 1.17E-67 | up |
| VIT_01s0150g00120.t01 | 1245 | ethylene-responsive transcription factor ERF113 | 110 | 5.358214 | 15 | 1.095348 | 2.290362303 | 3.20E-12 | 4.91E-13 | up |
| VIT_14s0006g02290.t01 | 1189 | ethylene-responsive transcription factor ERF034 | 205 | 10.45608 | 29 | 2.217413 | 2.23739229 | 2.58E-21 | 2.30E-22 | up |
| VIT_00s0341g00070.t01 | 720 | ethylene-responsive transcription factor ERF038 | 244 | 20.552 | 49 | 6.187197 | 1.731920679 | 2.92E-18 | 3.04E-19 | up |
| VIT_16s0013g01070.t01 | 1077 | ethylene-responsive transcription factor ERF105 | 1532 | 86.26602 | 323 | 27.26573 | 1.661703413 | 7.26E-104 | 1.41E-105 | up |
| VIT_00s0632g00010.t01 | 692 | ethylene-responsive transcription factor ERF034 | 271 | 23.7498 | 62 | 8.145467 | 1.543845916 | 2.95E-17 | 3.23E-18 | up |
| VIT_16s0013g00970.t01 | 951 | ethylene-responsive transcription factor 5 | 2575 | 164.2077 | 672 | 64.24201 | 1.35393248 | 8.58E-129 | 1.34E-130 | up |
| VIT_16s0013g01000.t01 | 946 | ethylene-responsive transcription factor 5 | 1552 | 99.49409 | 415 | 39.88296 | 1.318838501 | 7.94E-75 | 2.07E-76 | up |
| VIT_16s0013g00990.t01 | 964 | ethylene-responsive transcription factor 5 | 2166 | 136.2631 | 614 | 57.90575 | 1.234615868 | 4.61E-94 | 9.86E-96 | up |
| VIT_16s0013g01090.t01 | 1027 | ethylene-responsive transcription factor 5 | 243 | 14.34936 | 69 | 6.108141 | 1.232181232 | 2.34E-11 | 3.85E-12 | up |
| VIT_00s0662g00040.t01 | 1456 | ethylene-responsive transcription factor RAP2-4 | 1043 | 43.44298 | 300 | 18.73226 | 1.213597937 | 1.77E-44 | 7.68E-46 | up |
| VIT_02s0025g01360.t01 | 795 | ethylene-responsive transcription factor ERF061 | 92 | 7.018065 | 29 | 3.316357 | 1.081474146 | 0.00031377 | 0.00011884 | up |
| VIT_05s0077g01860.t01 | 1089 | ethylene-responsive transcription factor RAP2-3 | 73 | 4.065291 | 104 | 8.682321 | -1.094721974 | 1.57E-06 | 4.20E-07 | down |
| VIT_07s0005g00820.t01 | 958 | ethylene-responsive transcription factor ERF073 | 227 | 14.37001 | 367 | 34.82819 | -1.27719458 | 8.6255E-26 | 6.3269E-27 | down |
| VIT_18s0001g08610.t01 | 2778 | ethylene-responsive transcription factor ANT | 111 | 2.42319 | 187 | 6.119835 | -1.336585408 | 1.53E-14 | 1.96E-15 | down |
| VIT_11s0016g00660.t01 | 957 | ethylene-responsive transcription factor ERF017 | 1406 | 89.09844 | 2932 | 278.5367 | -1.644395324 | 1.33E-292 | 1.027E-294 | down |
| VIT_02s0234g00130.t01 | 1123 | ethylene-responsive transcription factor 2 | 964 | 52.05878 | 2347 | 190.0044 | -1.867819605 | 1.09E-279 | 8.84E-282 | down |
| VIT_16s0100g00400.t01 | 979 | ethylene-responsive transcription factor ERF025 | 337 | 20.87584 | 924 | 85.80639 | -2.039251075 | 5.82E-124 | 9.53E-126 | down |
| VIT_03s0063g00460.t01 | 979 | ethylene-responsive transcription factor ERF109 | 739 | 45.77817 | 2262 | 210.0585 | -2.198059474 | 0 | 0 | down |
| VIT_07s0031g00720.t01 | 648 | ethylene-responsive transcription factor ERF109 | 63 | 5.896065 | 231 | 32.40913 | -2.458575933 | 1.87E-39 | 8.97E-41 | down |
| VIT_07s0031g00710.t01 | 604 | ethylene-responsive transcription factor ERF109 | 12 | 1.204872 | 54 | 8.128065 | -2.754031816 | 3.58E-11 | 5.98E-12 | down |
| VIT_16s0013g00890.t01 | 1108 | ethylene-responsive transcription factor 2 | 143 | 7.826958 | 777 | 63.75462 | -3.026006266 | 7.26E-163 | 9.30E-165 | down |
| **ABA** | | | | | | | | | | |
| VIT_15s0046g01050.t01 | 1128 | abscisic acid receptor PYL9 | 1839 | 98.87109 | 583 | 46.98831 | 1.073246877 | 4.89E-64 | 1.47E-65 | up |
| VIT_08s0058g00470.t01 | 866 | abscisic acid receptor PYL4 | 33 | 2.310962 | 80 | 8.398514 | -1.86164079 | 2.1945E-10 | 3.9125E-11 | down |
| VIT_07s0129g00520.t01 | 1445 | protein phosphatase 2C 63 (PP2c) | 183 | 7.680331 | 250 | 15.72905 | -1.034191261 | 4.82E-13 | 6.93E-14 | down |
| VIT_01s0011g03910.t01 | 1815 | protein phosphatase 2C 15 | 238 | 7.952378 | 334 | 16.73016 | -1.072993344 | 4.40E-18 | 4.62E-19 | down |
| VIT_05s0020g02940.t01 | 1800 | protein phosphatase 2C 70 | 103 | 3.470255 | 150 | 7.57616 | -1.126424978 | 2.56E-09 | 5.03E-10 | down |
| VIT_07s0005g02110.t01 | 1603 | protein phosphatase 2C 49 | 1591 | 60.19125 | 2431 | 137.8738 | -1.195722872 | 4.20E-150 | 5.85E-152 | down |
| VIT_12s0028g03310.t01 | 1109 | protein phosphatase 2C 58 | 130 | 7.109 | 201 | 16.47764 | -1.212790693 | 1.63E-13 | 2.26E-14 | down |
| VIT_19s0015g01920.t01 | 2377 | protein phosphatase 2C 6 | 94 | 2.398255 | 147 | 5.622358 | -1.229190308 | 2.32E-10 | 4.15E-11 | down |
| VIT_18s0001g09390.t01 | 1530 | protein phosphatase 2C 63 | 138 | 5.469963 | 223 | 13.25085 | -1.276482258 | 4.88E-16 | 5.70E-17 | down |
| VIT_08s0007g00310.t01 | 2923 | protein phosphatase 2C 23 | 214 | 4.439987 | 401 | 12.47228 | -1.490098255 | 2.712E-35 | 1.4775E-36 | down |
| **JA** | | | | | | | | | | |
| VIT_18s0041g02080.t01 | 1314 | 12-oxophytodienoate reductase 2 | 169 | 7.799882 | 470 | 32.51868 | -2.059744325 | 3.42E-64 | 1.02E-65 | down |
| VIT_18s0041g02020.t01 | 1364 | 12-oxophytodienoate reductase 2 | 8 | 0.355691 | 94 | 6.265329 | -4.138695666 | 9.85E-26 | 7.26E-27 | down |
| VIT_10s0003g03790.t01 | 622 | protein TIFY 5A (JAZ) | 111 | 10.82254 | 158 | 23.09389 | -1.093471696 | 2.37E-09 | 4.64E-10 | down |
| VIT_10s0003g03800.t01 | 684 | protein TIFY 5A (JAZ) | 81 | 7.181673 | 118 | 15.68398 | -1.126899861 | 1.46E-07 | 3.45E-08 | down |
| VIT_01s0011g05560.t01 | 1622 | protein TIFY 6B, transcript variant 1 | 1528 | 57.13066 | 2320 | 130.0372 | -1.186587077 | 1.54E-141 | 2.21E-143 | down |
| VIT_17s0000g02230.t01 | 1562 | protein TIFY 6B | 2228 | 86.50294 | 3384 | 196.9608 | -1.18708715 | 2.29E-206 | 2.39E-208 | down |
| VIT_09s0002g00890.t01 | 1117 | protein TIFY 10A | 230 | 12.48738 | 452 | 36.7888 | -1.558795726 | 2.45E-42 | 1.12E-43 | down |
| VIT_11s0016g00710.t01 | 1284 | protein TIFY 10A | 1291 | 60.97586 | 2742 | 194.1479 | -1.670846385 | 5.88E-280 | 4.76E-282 | down |
| VIT_10s0003g03810.t01 | 521 | protein TIFY 5A (JAZ) | 536 | 62.39126 | 1187 | 207.1302 | -1.731121844 | 1.14E-127 | 1.80E-129 | down |
| **GA** | | | | | | | | | | |
| VIT_14s0108g00740.t01 | 700 | gibberellin-regulated protein 4 | 28713 | 2487.581 | 6446 | 837.1873 | 1.571121166 | 0 | 0 | up |
| VIT_17s0000g06210.t01 | 620 | gibberellin-regulated protein 6 | 499 | 48.80963 | 150 | 21.9953 | 1.1499705 | 4.87E-20 | 4.59E-21 | up |
| VIT_14s0030g00440.t01 | 1574 | gibberellin receptor GID1A (GID1A) | 1331 | 51.2826 | 440 | 25.41431 | 1.012828328 | 2.01E-42 | 9.17E-44 | up |
| VIT_14s0066g01790.t01 | 1104 | gibberellin-regulated protein 14 | 46 | 2.526885 | 78 | 6.423266 | -1.345947077 | 9.5832E-07 | 2.4975E-07 | down |
| VIT_19s0014g04940.t01 | 2490 | chitin-inducible gibberellin-responsive protein 1 | 939 | 22.86983 | 1802 | 65.79393 | -1.524508763 | 2.44E-161 | 3.19E-163 | down |
| VIT_19s0140g00120.t01 | 1223 | gibberellin 2-beta-dioxygenase 1 | 78 | 3.867807 | 295 | 21.92936 | -2.50327574 | 3.47E-51 | 1.29E-52 | down |

**Additional file 9** Differently-expressed genes related to Ion Transport

| #Gene | Length | Function | read_A | RPKM_A | read_B | RPKM_B | log2 | q-value | p_value | Result |
| --- | --- | --- | --- | --- | --- | --- | --- | --- | --- | --- |
| **V-type proton ATPase** | | | | | | | | | | |
| VIT_04s0008g04760.t01 | 1024 | V-type proton ATPase 21 kDa proteolipid subunit | 1955 | 115.7827 | 646 | 57.3539 | 1.013455723 | 5.36E-62 | 1.66E-63 | up |
| VIT_08s0056g00620.t01 | 937 | V-type proton ATPase subunit E1 | 216 | 13.98012 | 293 | 28.42879 | -1.023976167 | 7.06E-15 | 8.85E-16 | down |
| **sodium/hydrogen exchanger** | | | | | | | | | | |
| VIT_14s0128g00020.t01 | 1718 | sodium/hydrogen exchanger 2 | 83 | 2.929892 | 214 | 11.32455 | -1.95053437 | 1.04E-27 | 7.19E-29 | down |
| VIT_15s0024g00260.t01 | 883 | sodium/hydrogen exchanger 6 | 46 | 3.159322 | 109 | 11.22267 | -1.828729183 | 1.83E-13 | 2.55E-14 | down |
| VIT_05s0020g01960.t01 | 2124 | nhx1 antiporter | 262 | 7.480721 | 380 | 16.2652 | -1.120539421 | 7.48E-22 | 6.48E-23 | down |
| CNGC VIT_06s0004g02670.t01 | 2708 | putative cyclic nucleotide-gated ion channel 15 | 312 | 10.47457 | 219 | 4.904471 | 1.094721974 | 1.75E-17 | 1.89E-18 | up |
|  |  |  |  |  |  |  |  |  |  |  |
| VIT_04s0069g00790.t01 | 2452 | probable cyclic nucleotide-gated ion channel 14 | 168 | 6.229012 | 117 | 2.893757 | 1.106059518 | 4.83E-10 | 8.90E-11 | up |
| VIT_11s0065g00930.t01 | 1552 | probable cyclic nucleotide-gated ion channel 17 | 121 | 4.72814 | 190 | 11.12993 | -1.235099186 | 3.55E-13 | 5.06E-14 | down |
| **potassium transporter** | | | | | | | | | | |
| VIT_14s0068g00850.t01 | 2624 | potassium transporter 4 | 2967 | 68.57257 | 4192 | 145.2405 | -1.082740605 | 1.76E-220 | 1.72E-222 | down |
| VIT_01s0011g06560.t01 | 2342 | potassium transporter 17 | 171 | 4.427983 | 345 | 13.39253 | -1.596706851 | 1.20E-33 | 6.94E-35 | down |
| VIT_11s0016g02400.t01 | 2174 | potassium efflux antiporter 4 | 268 | 7.476046 | 371 | 15.51475 | -1.053293001 | 2.08E-19 | 2.02E-20 | down |
| VIT_16s0022g02060.t01 | 2188 | potassium efflux antiporter 5 | 198 | 5.488006 | 278 | 11.55122 | -1.073691267 | 3.14E-15 | 3.85E-16 | down |
| **sodium-related cotransporter** | | | | | | | | | | |
| VIT_11s0016g04560.t01 | 1583 | sodium/pyruvate cotransporter BASS2, chloroplastic | 132 | 5.056962 | 210 | 12.0606 | -1.253958213 | 8.97493E-15 | 1.1332E-15 | down |
| VIT_13s0106g00740.t01 | 1479 | sodium/bile acid cotransporter 7 | 107 | 4.387451 | 205 | 12.60132 | -1.522119928 | 4.69E-19 | 4.64E-20 | down |

**Additional file 10** Differently-expressed genes related to Transcription factors

| #Gene | Length | | Function | read_A | RPKM_A | read_B | RPKM_B | log2 | q-value | p_value | Result |
| --- | --- | --- | --- | --- | --- | --- | --- | --- | --- | --- | --- |
| **CHCH** | | | | | | | | | | | |
| VIT_08s0007g08360.t01 | 1216 | | zinc finger AN1 and C2H2 domain | 62 | 3.092109 | 124 | 9.270827 | -1.58410681 | 1.38E-12 | 5 2.06E-13 | down |
| **dof** | | | | | | | | | | | |
| VIT_00s0652g00010.t01 | 1643 | | dof zinc finger protein DOF1.1 | 446 | 16.46243 | 85 | 4.703398 | 1.807402149 | 1.57E-34 | 8.73E-36 | up |
| VIT_01s0026g02580.t01 | 1967 | | dof zinc finger protein DOF5.2 | 297 | 9.156907 | 65 | 3.004273 | 1.607844493 | 6.69E-20 | 6.34E-21 | up |
| VIT_06s0004g03420.t01 | 1069 | | dof zinc finger protein DOF2.1 | 432 | 24.50771 | 114 | 9.695217 | 1.337890673 | 6.61E-22 | 5.70E-23 | up |
| VIT_02s0025g02250.t01 | 1539 | | dof zinc finger protein DOF4.6 | 81 | 3.191855 | 153 | 9.038226 | -1.501644654 | 3.28E-14 | 4.33E-15 | down |
| **ERF** | | | | | | | | | | | |
| VIT_05s0049g00510.t01 | 1264 | | ethylene-responsive transcription factor 1B | 221 | 10.60332 | 8 | 0.575405 | 4.203795745 | 3.43E-40 | 1.63E-41 | up |
| VIT_18s0001g10150.t01 | 780 | | ethylene-responsive transcription factor ERF010 | 534 | 41.51866 | 56 | 6.527153 | 2.669234195 | 4.02E-66 | 1.17E-67 | up |
| VIT_01s0150g00120.t01 | 1245 | | ethylene-responsive transcription factor ERF113 | 110 | 5.358214 | 15 | 1.095348 | 2.290362303 | 3.20E-12 | 4.91E-13 | up |
| VIT_14s0006g02290.t01 | 1189 | | ethylene-responsive transcription factor ERF034 | 205 | 10.45608 | 29 | 2.217413 | 2.23739229 | 2.58E-21 | 2.30E-22 | up |
| VIT_00s0341g00070.t01 | 720 | | ethylene-responsive transcription factor ERF038 | 244 | 20.552 | 49 | 6.187197 | 1.731920679 | 2.92E-18 | 3.04E-19 | up |
| VIT_16s0013g01070.t01 | 1077 | | ethylene-responsive transcription factor ERF105 | 1532 | 86.26602 | 323 | 27.26573 | 1.661703413 | 7.26E-104 | 1.41E-105 | up |
| VIT_00s0632g00010.t01 | 692 | | ethylene-responsive transcription factor ERF034 | 271 | 23.7498 | 62 | 8.145467 | 1.543845916 | 2.95E-17 | 3.23E-18 | up |
| VIT_16s0013g00970.t01 | 951 | | ethylene-responsive transcription factor 5 | 2575 | 164.2077 | 672 | 64.24201 | 1.35393248 | 8.58E-129 | 1.34E-130 | up |
| VIT_16s0013g01000.t01 | 946 | | ethylene-responsive transcription factor 5 | 1552 | 99.49409 | 415 | 39.88296 | 1.318838501 | 7.94E-75 | 2.07E-76 | up |
| VIT_16s0013g00990.t01 | 964 | | ethylene-responsive transcription factor 5 | 2166 | 136.2631 | 614 | 57.90575 | 1.234615868 | 4.61E-94 | 9.86E-96 | up |
| VIT_16s0013g01090.t01 | 1027 | | ethylene-responsive transcription factor 5 | 243 | 14.34936 | 69 | 6.108141 | 1.232181232 | 2.34E-11 | 3.85E-12 | up |
| VIT_00s0662g00040.t01 | 1456 | | ethylene-responsive transcription factor RAP2-4 | 1043 | 43.44298 | 300 | 18.73226 | 1.213597937 | 1.77E-44 | 7.68E-46 | up |
| VIT_02s0025g01360.t01 | 795 | | ethylene-responsive transcription factor ERF061 | 92 | 7.018065 | 29 | 3.316357 | 1.081474146 | 0.000314 | 0.000119 | up |
| VIT_05s0077g01860.t01 | 1089 | | ethylene-responsive transcription factor RAP2-3 | 73 | 4.065291 | 104 | 8.682321 | -1.09472197 | 1.57E-06 | 4.20E-07 | down |
| VIT_07s0005g00820.t01 | 958 | | ethylene-responsive transcription factor ERF073 | 227 | 14.37001 | 367 | 34.82819 | -1.27719458 | 8.63E-26 | 6.33E-27 | down |
| VIT_18s0001g08610.t01 | 2778 | | AP2 ethylene-responsive transcription factor ANT | 111 | 2.42319 | 187 | 6.119835 | -1.33658540 | 1.53E-14 | 1.96E-15 | down |
| VIT_18s0001g08610.t01 | 2778 | | ethylene-responsive transcription factor ANT | 111 | 2.42319 | 187 | 6.119835 | -1.33658540 | 1.53E-14 | 1.96E-15 | down |
| VIT_11s0016g00660.t01 | 957 | | ethylene-responsive transcription factor ERF017 | 1406 | 89.09844 | 2932 | 278.5367 | -1.64439532 | 1.3E-292 | 1E-294 | down |
| VIT_02s0234g00130.t01 | 1123 | | ethylene-responsive transcription factor 2 | 964 | 52.05878 | 2347 | 190.0044 | -1.867819605 | 1.09E-279 | 8.84E-282 | down |
| VIT_16s0100g00400.t01 | 979 | | ethylene-responsive transcription factor ERF025 | 337 | 20.87584 | 924 | 85.80639 | -2.039251075 | 5.82E-124 | 9.53E-126 | down |
| VIT_03s0063g00460.t01 | 979 | | ethylene-responsive transcription factor ERF109 | 739 | 45.77817 | 2262 | 210.0585 | -2.198059474 | 0 | 0 | down |
| VIT_07s0031g00720.t01 | 648 | | ethylene-responsive transcription factor ERF109 | 63 | 5.896065 | 231 | 32.40913 | -2.458575933 | 1.87E-39 | 8.97E-41 | down |
| VIT_07s0031g00710.t01 | 604 | | ethylene-responsive transcription factor ERF109 | 12 | 1.204872 | 54 | 8.128065 | -2.754031816 | 3.58E-11 | 5.98E-12 | down |
| VIT_16s0013g00890.t01 | 1108 | | ethylene-responsive transcription factor 2 | 143 | 7.826958 | 777 | 63.75462 | -3.026006266 | 7.26E-163 | 9.30E-165 | down |
| **homeobox-leucine zipper** | | | | | | | | | | | |
| VIT_14s0108g00390.t01 | 852 | | homeobox-leucine zipper protein HOX21 | 119 | 8.470403 | 18 | 1.920717 | 2.140785947 | 3.60E-12 | 5.55E-13 | up |
| VIT_00s0299g00100.t01 | 1356 | | homeobox-leucine zipper protein HAT4 | 1107 | 49.50905 | 243 | 16.2921 | 1.603520189 | 1.62E-71 | 4.40E-73 | up |
| VIT_08s0007g06670.t01 | 1035 | | homeobox-leucine zipper protein HAT14 | 106 | 6.21101 | 28 | 2.459507 | 1.336458718 | 3.70E-06 | 1.04E-06 | up |
| VIT_17s0000g05630.t01 | 1518 | | homeobox-leucine zipper protein HAT5 | 2294 | 91.64702 | 633 | 37.91074 | 1.273481172 | 1.68E-104 | 3.25E-106 | up |
| VIT_15s0021g01880.t01 | 1046 | | homeobox-leucine zipper protein HAT3 | 948 | 54.96337 | 264 | 22.94577 | 1.260242315 | 6.30E-43 | 2.85E-44 | up |
| **NAC** | | | | | | | | | | | |
| VIT_19s0014g03290.t01 | 1440 | | NAC domain-containing protein 72 | 161 | 6.780474 | 49 | 3.093599 | 1.132100219 | 5.05E-07 | 1.27E-07 | up |
| VIT_11s0016g02880.t01 | 2099 | | NAC domain-containing protein 8 | 88 | 2.542535 | 124 | 5.370808 | -1.078871506 | 1.99E-07 | 4.77E-08 | down |
| VIT_18s0001g02300.t01 | 1299 | | NAC domain-containing protein 2 | 1097 | 51.21465 | 1992 | 139.4153 | -1.444760936 | 1.18E-164 | 1.50E-166 | down |
| VIT_07s0031g02610.t01 | 1467 | | NAC domain-containing protein 2 | 1362 | 56.30458 | 4717 | 292.3251 | -2.376249712 | 0 | 0 | down |
| VIT_06s0080g00780.t01 | 991 | | NAC domain-containing protein 90 | 32 | 1.958272 | 144 | 13.2105 | -2.754031816 | 4.02E-28 | 2.74E-29 | down |
| VIT_06s0004g00020.t01 | 1079 | | NAC domain-containing protein 68 | 0.5 | 0.028103 | 63 | 5.308227 | -7.561386738 | 6.90E-18 | 7.33E-19 | down |
| **PHD** | | | | | | | | | | | |
| VIT_18s0089g01050.t01 | 2223 | | PHD finger protein | 297 | 8.1024 | 84 | 3.435344 | 1.237894883 | 9.74E-14 | 1.33E-14 | up |
| VIT_05s0062g01360.t01 | 522 | | PHD finger | 263 | 30.55498 | 57 | 9.927382 | 1.621922161 | 5.96E-18 | 6.32E-19 | up |
| VIT_11s0016g01510.t01 | 1511 | | PHD finger protein ALFIN 5 | 192 | 7.706079 | 33 | 1.985546 | 1.956461567 | 4.96E-17 | 5.48E-18 | up |
| **WRKY** | | | | | | | | | | | |
| VIT_07s0031g01710.t01 | 571 | | WRKY transcription factor 51 | 104 | 11.04572 | 301 | 47.92485 | -2.117286773 | 7.47E-43 | 3.38E-44 | down |
| VIT_04s0008g05760.t01 | 1174 | | WRKY3 transcription factor | 1009 | 52.12184 | 2749 | 212.8811 | -2.030087547 | 0 | 0 | down |
| VIT_09s0018g00240.t01 | 1355 | | WRKY4 transcription factor | 172 | 7.698141 | 441 | 29.58896 | -1.942476905 | 6.93E-56 | 2.36E-57 | down |
| VIT_10s0116g01200.t01 | 1940 | | WRKY transcription factor 6 | 862 | 26.94649 | 2042 | 95.69393 | -1.828329906 | 1.93E-236 | 1.80E-238 | down |
| VIT_08s0058g00690.t01 | 1904 | | WRKY transcription factor 33 | 88 | 2.802931 | 170 | 8.117314 | -1.534066132 | 3.82E-16 | 4.44E-17 | down |
| VIT_08s0058g01390.t01 | 1175 | | WRKY transcription factor 70 | 1122 | 57.90975 | 2009 | 155.4435 | -1.424511703 | 1.39E-162 | 1.79E-164 | down |
| VIT_14s0108g01280.t01 | 2202 | | WRKY transcription factor 4 | 281 | 7.739015 | 441 | 18.20756 | -1.23431534 | 2.98E-29 | 1.93E-30 | down |
| VIT_06s0004g07500.t01 | 2001 | | WRKY transcription factor 33 | 709 | 21.48799 | 986 | 44.79816 | -1.059908834 | 1.35E-50 | 5.14E-52 | down |
| **bHLH** | | | | | | | | | | | |
| VIT_14s0108g00420.t01 | 568 | transcription factor bHLH135 | | 490 | 52.31719 | 111 | 17.76663 | 1.558115258 | 7.78E-31 | 4.84E-32 | up |
| VIT_11s0052g00100.t01 | 934 | transcription factor bHLH35 | | 707 | 45.90598 | 951 | 92.56867 | -1.011841941 | 2.64E-45 | 1.12E-46 | down |
| VIT_10s0003g02950.t01 | 472 | transcription factor bHLH144, transcript variant 1 | | 53 | 6.809741 | 89 | 17.14267 | -1.331919791 | 1.93E-07 | 4.62E-08 | down |
| VIT_12s0028g03550.t01 | 906 | transcription factor bHLH118 | | 77 | 5.154176 | 193 | 19.36687 | -1.909777311 | 2.10E-24 | 1.62E-25 | down |
| VIT_05s0020g04780.t01 | 1057 | transcription factor bHLH92 | | 95 | 5.450613 | 349 | 30.01793 | -2.461334432 | 2.80E-59 | 9.07E-61 | down |
| **MYB** |  |  | |  |  |  |  |  |  |  |  |
| VIT_16s0050g02530.t01 | 550 | Myb-related TRIPTYCHON protein (TRY) | | 58 | 6.395316 | 11 | 1.818278 | 1.814442562 | 2.04E-05 | 6.33E-06 | up |
| VIT_10s0116g01760.t01 | 345 | transcription repressor MYB4 | | 123 | 21.62135 | 24 | 6.324446 | 1.77344519 | 5.21E-10 | 9.63E-11 | up |
| VIT_04s0023g03710.t01 | 883 | R2R3 MYB 4b transcription factor (MYB4B) | | 333 | 22.87074 | 68 | 7.001298 | 1.707808711 | 4.21E-24 | 3.29E-25 | up |
| VIT_14s0006g01620.t01 | 1361 | transcription repressor MYB6 | | 218 | 9.713932 | 51 | 3.406767 | 1.511652168 | 1.11E-13 | 1.52E-14 | up |
| VIT_17s0000g02660.t01 | 909 | transcription repressor MYB6 (MYBC2-L2) | | 41 | 2.735374 | 67 | 6.701026 | -1.292644 | 1.19E-05 | 3.59E-06 | down |
| **ZAT** | | | | | | | | | | | |
| VIT_08s0007g04770.t01 | 1030 | | zinc finger protein ZAT5 | 152 | 8.949588 | 49 | 4.325031 | 1.049110855 | 4.89E-06 | 1.39E-06 | up |
| VIT_00s0229g00050.t01 | 1079 | | zinc finger protein ZAT4 | 47 | 2.641637 | 71 | 5.982287 | -1.179265082 | 2.86E-05 | 9.08E-06 | down |
| VIT_07s0129g00240.t01 | 1128 | | zinc finger protein ZAT10 | 80 | 4.301081 | 132 | 10.63886 | -1.306572839 | 3.01E-10 | 5.45E-11 | down |
| VIT_03s0091g00690.t01 | 925 | | zinc finger protein ZAT10 | 268 | 17.57073 | 532 | 52.28779 | -1.57330006 | 2.40E-50 | 9.14E-52 | down |

**Additional file 11** Primers used for qRT-PCR

| Gene | Description | Forward Primer (5´to 3´) | Reverse Primer (5´to 3´) |
| --- | --- | --- | --- |
| VIT_04s0023g00530.t01 | auxin-induced protein 15A | ATCAGCAACAACAGCAGTGG | TGCATGGGATTGTGACTCCA |
| VIT_05s0020g03740.t01 | non-specific lipid-transfer protein-like protein | TAAGAGTGACTGGCATGGGG | GGCTAGTTGTGAGCAGCATG |
| VIT_05s0049g01070.t01 | probable glutathione S-transferase-like | ACACCCTTATGAACGAGCCA | CAACAGCCTCTATCGCCAAC |
| VIT_05s0049g00520.t01 | proline-rich cell wall protein-like (GRIP4) | GTCTTCTGCTTGCTCACTCG | CAGGAAGAGGTGGTTTGTGC |
| VIT_19s0015g01070.t01 | calcineurin B-like protein 02 (CBL02) | TTGAGAGGCAGGAGGTGAAG | TCAAAAGGGATGGGTGTCGT |
| VIT_14s0128g00020.t01 | sodium/hydrogen exchanger 2-like | AACTGACATTGCAAGCCTGG | GGGTATCTCATCAGCAGCCT |
| VIT_00s0332g00110.t01 | Photosystem II reaction center protein J | GGGGTTGGTTATTCGTCAGC | TTCCATTCCTTGTCGGCTCT |
| VIT_00s0201g00080.t01 | probable WRKY transcription factor 33-like | ACTAGCACAGACACTACCCA | TGTTGCCTTAAATGGTGCCTTAT |
| VIT_18s0001g08590.t01 | Putative uncharacterized protein | TGGCCCCATCTGAGTATTGG | ATTCCTCCGTCAGCAGTCTT |
| VIT_11s0052g01180.t01 | probable xyloglucan endotransglucosylase/ hydrolase protein 23-like | GAAACTCTGCTGGCACTGTC | CTTGCCTTGGCTGAACACAT |
| VIT_13s0067g02360.t01 | peroxidase 4-like | CGCTCCTACGCCTTTTCTTC | GAACATCAGCACACGAGACC |
| VIT_16s0050g02530.t01 | Myb-related tripcription-like protein (TRY) | TCGCAGTCTGAAGAGGTGAG | TTTCCCCTCTCAGCATACCC |
| VIT_12s0035g01910.t01 | 22.0 kDa class IV heat shock protein-like | ACCCTCAAAGTCTCCTCTGC | TTCTGGTGTAGGGCATGAGG |
| VIT_06s0004g05670.t01 | glutathione S-transferase U7-like | GCTCTGGGAATGGAGATCCT | GCTGCCATTGATATGCCTTT |
| VIT_05s0062g00300.t01 | UDP-glycosyltransferase 75D1-like | AGCCAAGGAGAAGGGAGAAG | CACGAAACAACCCAGTGATG |
| VIT_07s0005g00160.t01 | alkylated DNA repair protein alkB homolog 8-like | GGGCAGGAAATTGATCAGAA | TGAAATTGGGATTGCAGACA |
| UBI | Housekeeping gene | GCTCGCTGTTTTGCAGTTCTAC | AACATAGGTGAGGCCGCACTT |
